# Supplementary material for: Catechol-Containing Schiff Bases on Thiacalixarene: Synthesis, Copper (II) Recognition, and Formation of Organic-Inorganic Copper-Based Materials
Source: Molecules. 2021 Apr 17;26(8):2334. doi: 10.3390/molecules26082334 (PMC8072794; doi:10.3390/molecules26082334)
Supplement: Supplementary file 1 [file molecules-26-02334-s001.zip › molecules-1186099-supplementary.pdf]

Supplementary Information

For

Catechol-Containing Schiff Bases on Thiacalixarene: Synthesis, Copper (II) Recognition and Formation of Organic-Inorganic Copper-based Materials

Pavel Padnya, Ksenia Shibaeva, Maxim Arsenyev, Svetlana Baryshnikova, Olga Terenteva, Igor Shiabiev, Artur Khannanov, Artur Boldyrev, Alexander Gerasimov, Denis Grishaev, Yurii Shtyrlin and Ivan Stoikov

Content

Table S1. Chemical shifts of the protons of compounds **8-11** in ^{1}H NMR spectra . . . 2

Figures S1-S4. ^{1}H NMR spectra of the compounds **8-11** 3-4

Figures S5-S8. ^{13}C NMR spectra of the compounds **8-11** 5-6

Figures S9-S12. FT-IR spectra of the compounds **8-11** 7-8

Figures S13-S16. HRMS spectra of the compounds **8-11** 9-10

Figures S17-S20. UV-vis spectra of the compounds **8-11** without/with d-metal cations . . 11-12

Figure S21-S24. Job's plots for the determination of the stoichiometry 13-14

Figures S25-S28. UV-vis spectra of the compounds **8-11** with Cu^{2+} cations . . . 15-18

Figures S29-S32. ^{1}H NMR spectra of the compounds **8-11** without/with Cu^{2+} cations . . 19-20

Figure S33. FT-IR spectra of the compound **10**, **10**+Cu mixture, **10**+Cu complex and $CuCl_2$. 21

Figure S34. Powder X-ray diffractograms of the compound **10**, $CuCl_2$, **10**+Cu mixture and **10**+Cu complex 22

Figure S35. TG curves of the compound **10** and **10**+Cu complex 22

Table S1. Chemical shifts (ppm) of the protons of compounds **8-11** in ^{1}H NMR spectra.

Compounds / protons	**8** (*cone*)	**9** (*partial cone*)	**10** (*1,3-alternate*)	**11** (monomer)
t-Bu	1.09 (s)	1.02 (s), 1.26 (s), 1.29 (s)	1.18 (s)	1.29 (s)
t-Bu	1.38 (s)	1.37 (s)	1.38 (s)	1.40 (s)
t-Bu	1.39 (s)	1.41 (s)	1.41 (s)	1.42 (s)
$NH(CH_2)_2(\underline{CH_2})_2(CH_2)_2N{=}CH$	1.35-1.42 (m)	1.42-1.48 (m)	1.35-1.43 (m)	1.33-1.48 (m)
$NHCH_2\underline{CH_2}(CH_2)_4N{=}CH$	1.59 (m)	1.59-1.67 (m)	1.60 (m)	1.56 (m)
$NH(CH_2)_4\underline{CH_2}CH_2N{=}CH$	1.70 (m)	1.72-1.76 (m)	1.74 (m)	1.72 (m)
$NH\underline{CH_2}(CH_2)_5N{=}CH$	3.33 (m)	3.27-3.42 (m)	3.24 (m)	3.34 (m)
$NH(CH_2)_5\underline{CH_2}N{=}CH$	3.56 (m)	3.61 (m)	3.61 (m)	3.58 (m)
OCH_2CO	4.80 (s)	4.26 (d of AB system), 4.86 (d of AB system), 4.44 (s), 4.96 (s),	4.06 (s)	4.46 (s)
$ArH(C_6H_1)$	6.52 (s)	6.53 (s)	6.60 (s)	6.56 (s)
ArH	7.33 (s)	7.05 (d of AB system), 7.45 (d of AB system), 7.61 (s), 7.76 (s),	7.52 (s)	6.85 and 7.33 (AA'BB' system)
NH	7.92 (br.t)	7.53 (br.t), 7.88 (br.t), 8.64 (br.t)	7.75 (br.t)	6.63 (br.t)
CH=N	8.80 (s)	8.83 (s)	8.84 (s)	8.82 (s)

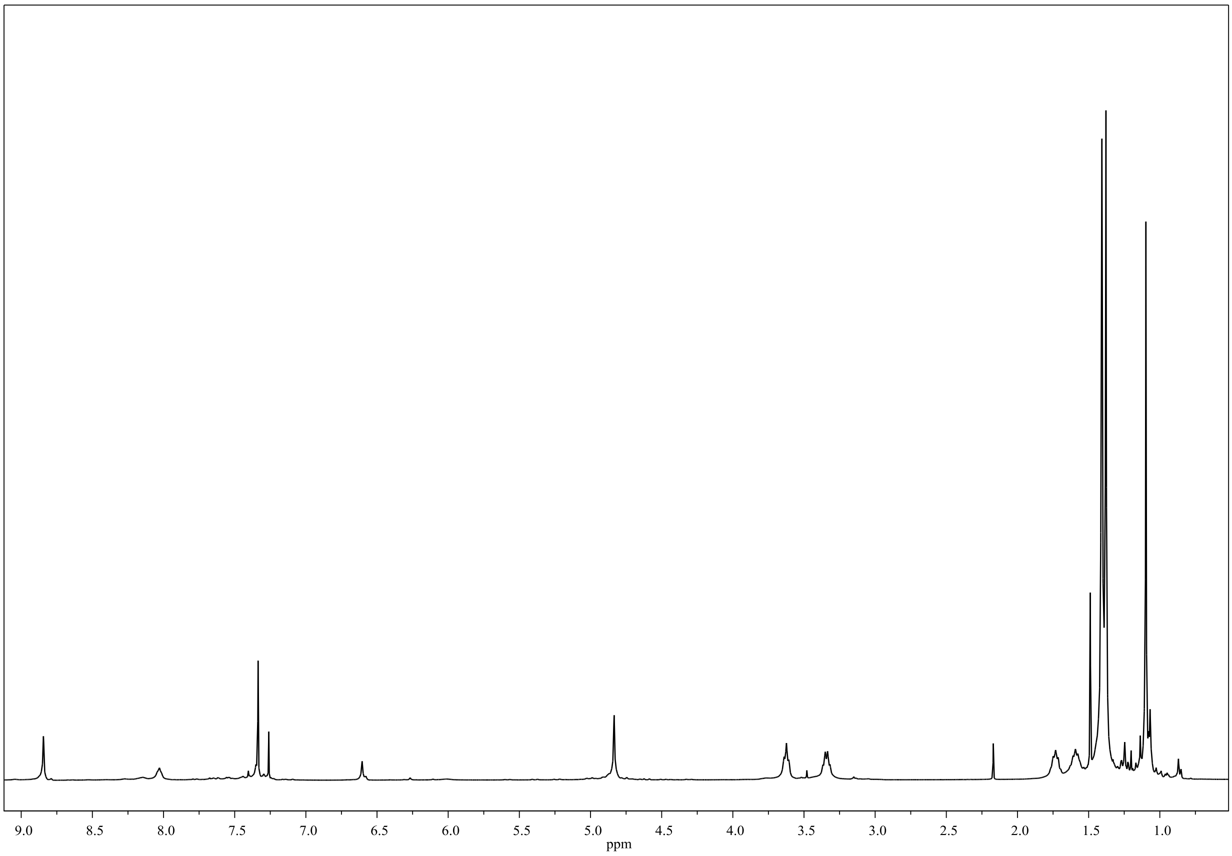

Figure S1. ¹H NMR spectrum of the compound **8** (*cone*), $CDCl_3$, 298 K, 400 MHz.

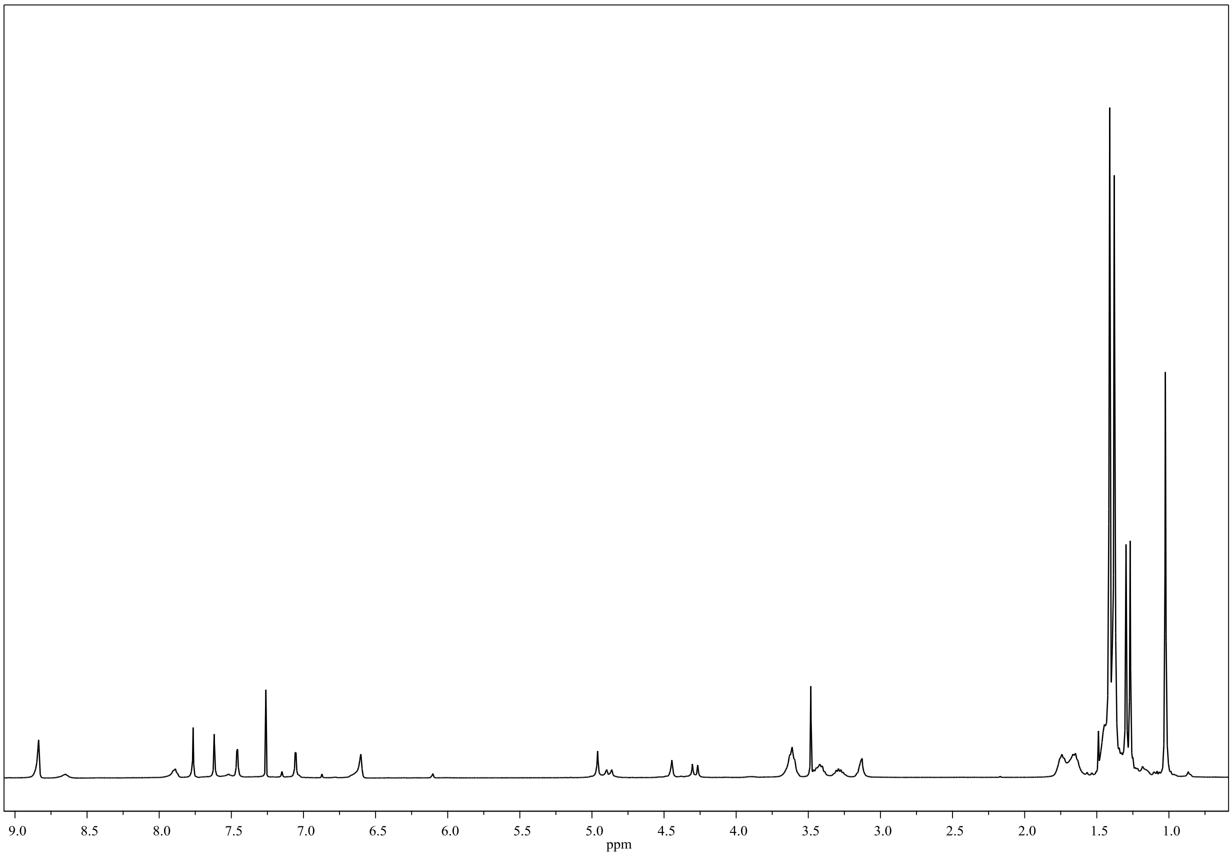

Figure S2. ¹H NMR spectrum of the compound **9** (*partial cone*), $CDCl_3$, 298 K, 400 MHz.

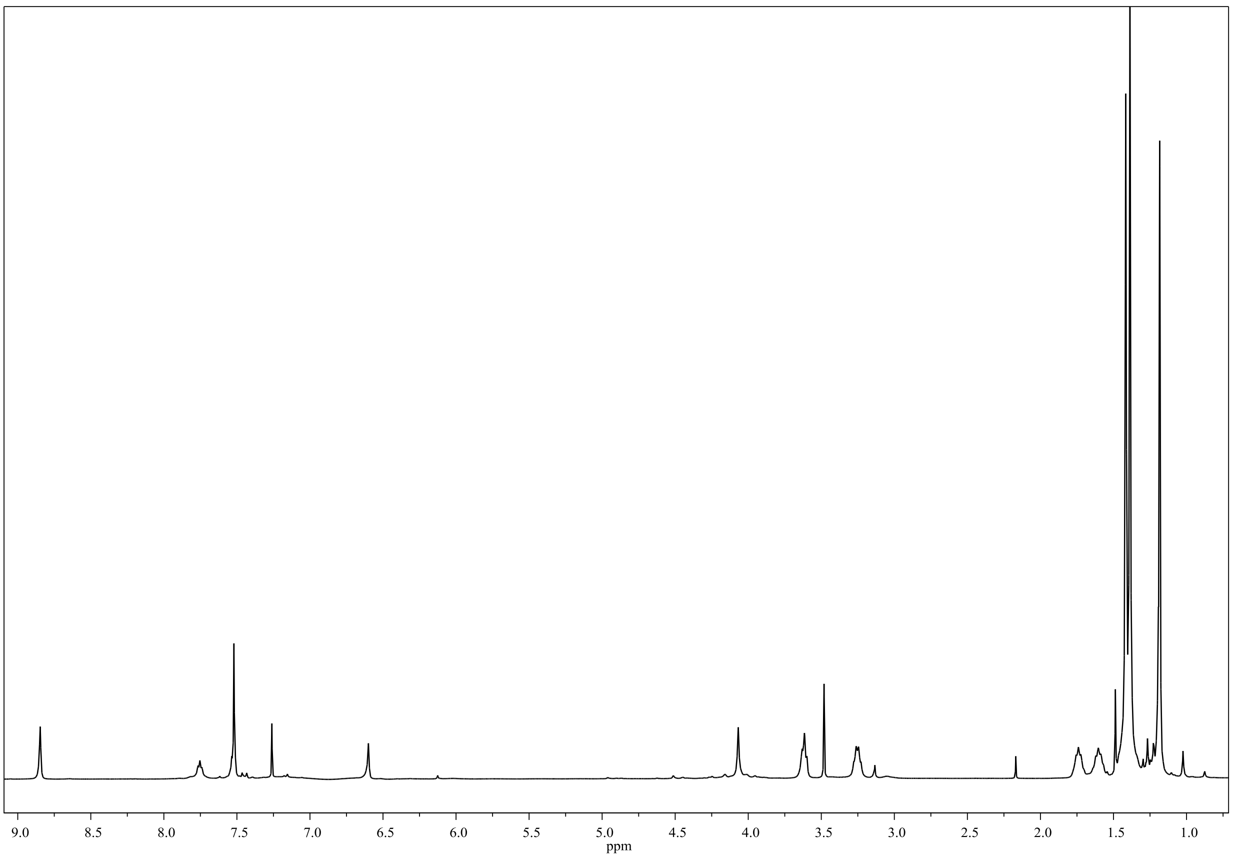

Figure S3. ^{1}H NMR spectrum of the compound **10** (*1,3-alternate*), $CDCl_3$, 298 K, 400 MHz.

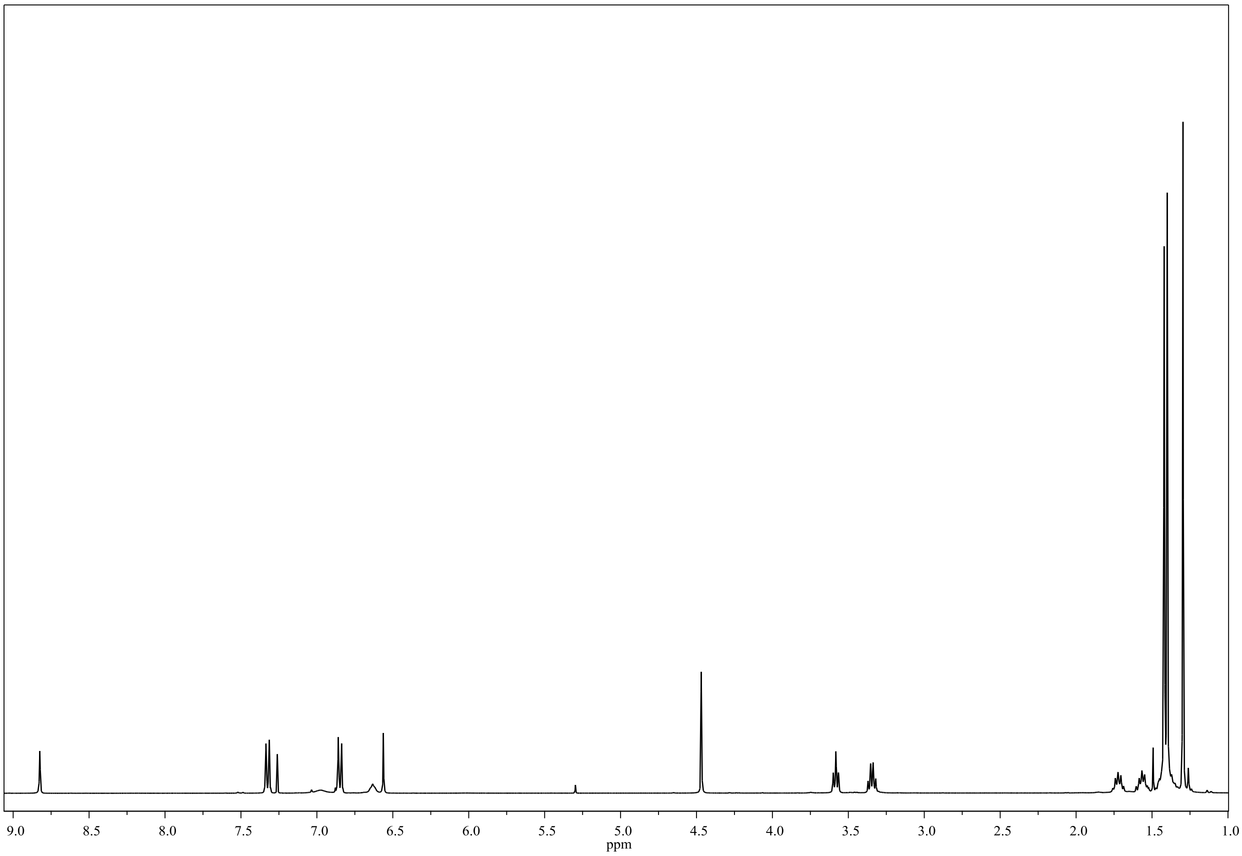

Figure S4. ^{1}H NMR spectrum of the compound **11** (*monomer*), $CDCl_3$, 298 K, 400 MHz.

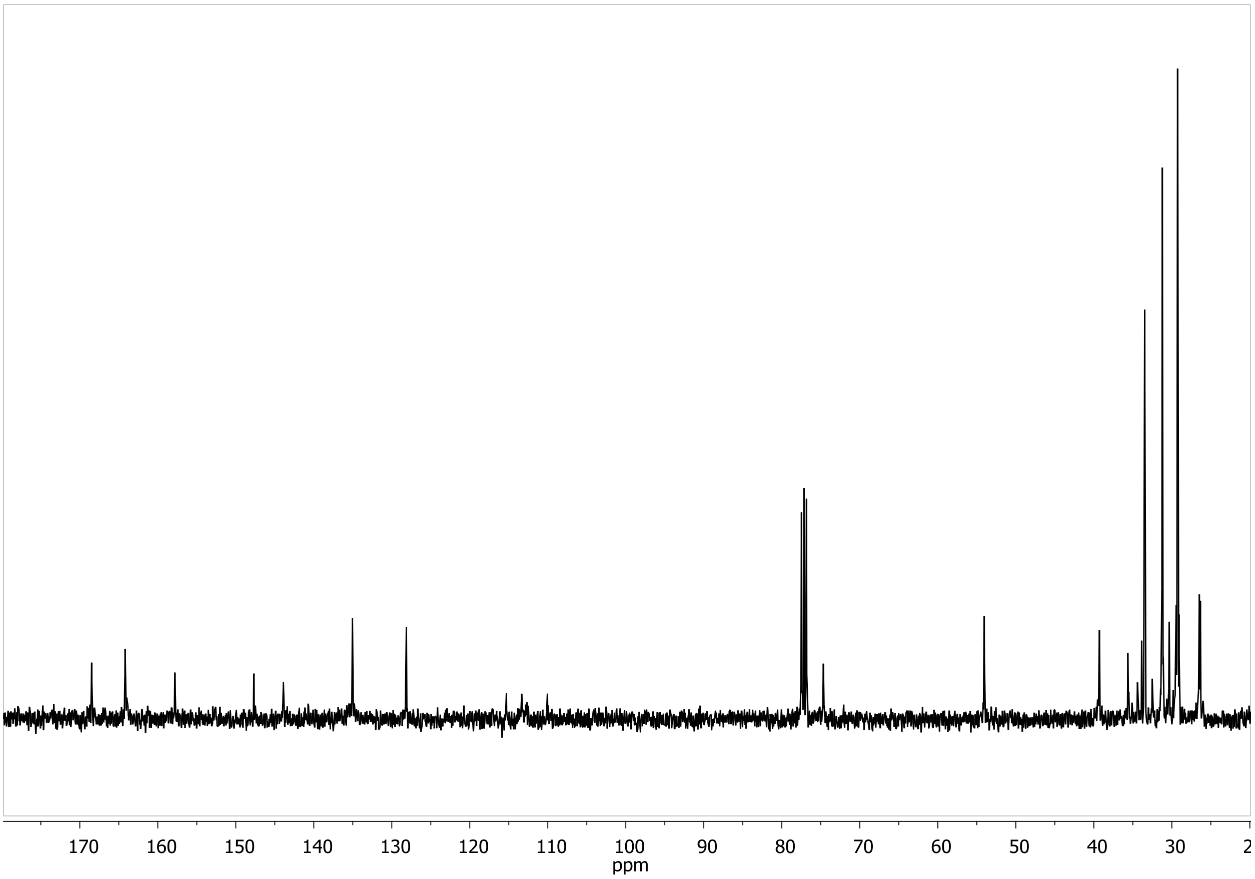

Figure S5. ^{13}C NMR spectrum of the compound **8** (*cone*), $CDCl_3$, 298 K, 100 MHz.

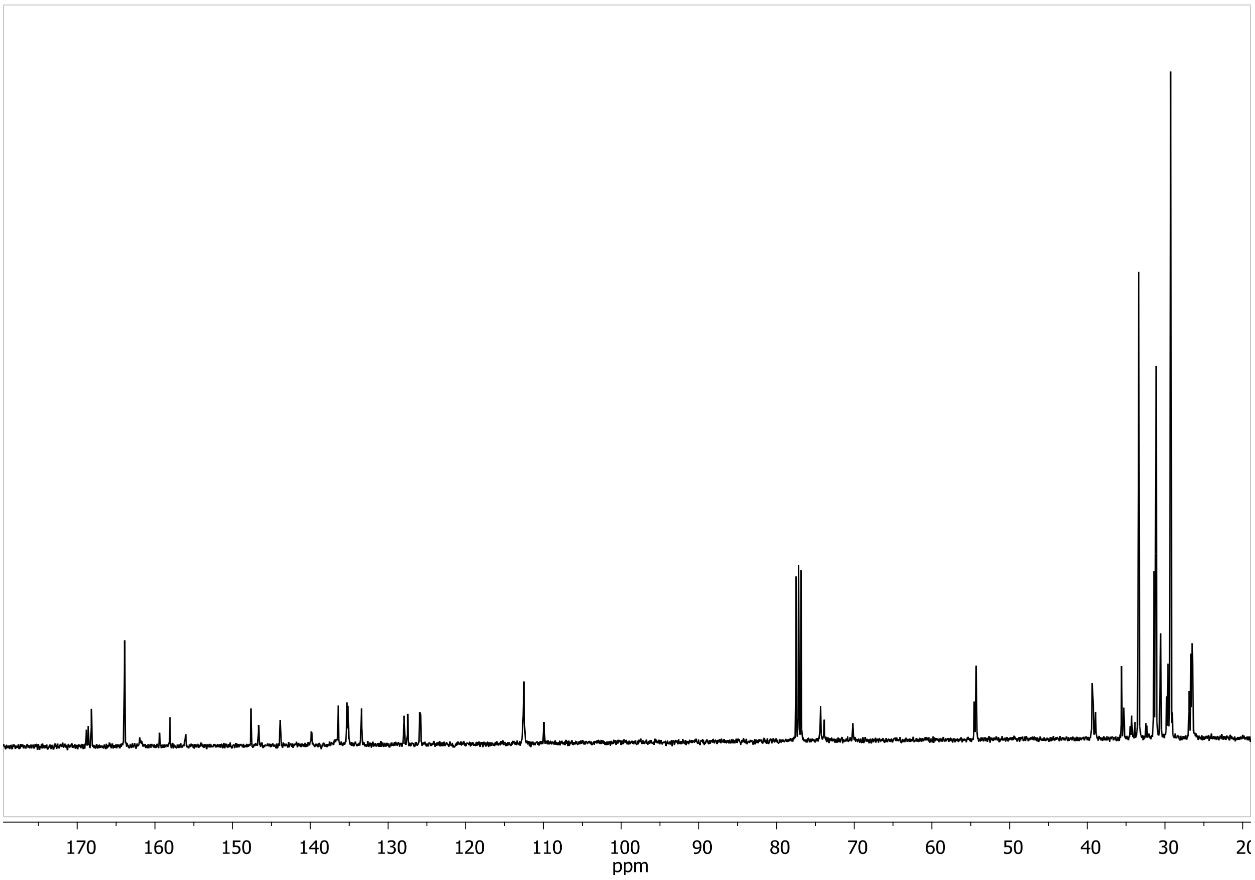

Figure S6. ^{13}C NMR spectrum of the compound **9** (*partial cone*), $CDCl_3$, 298 K, 100 MHz.

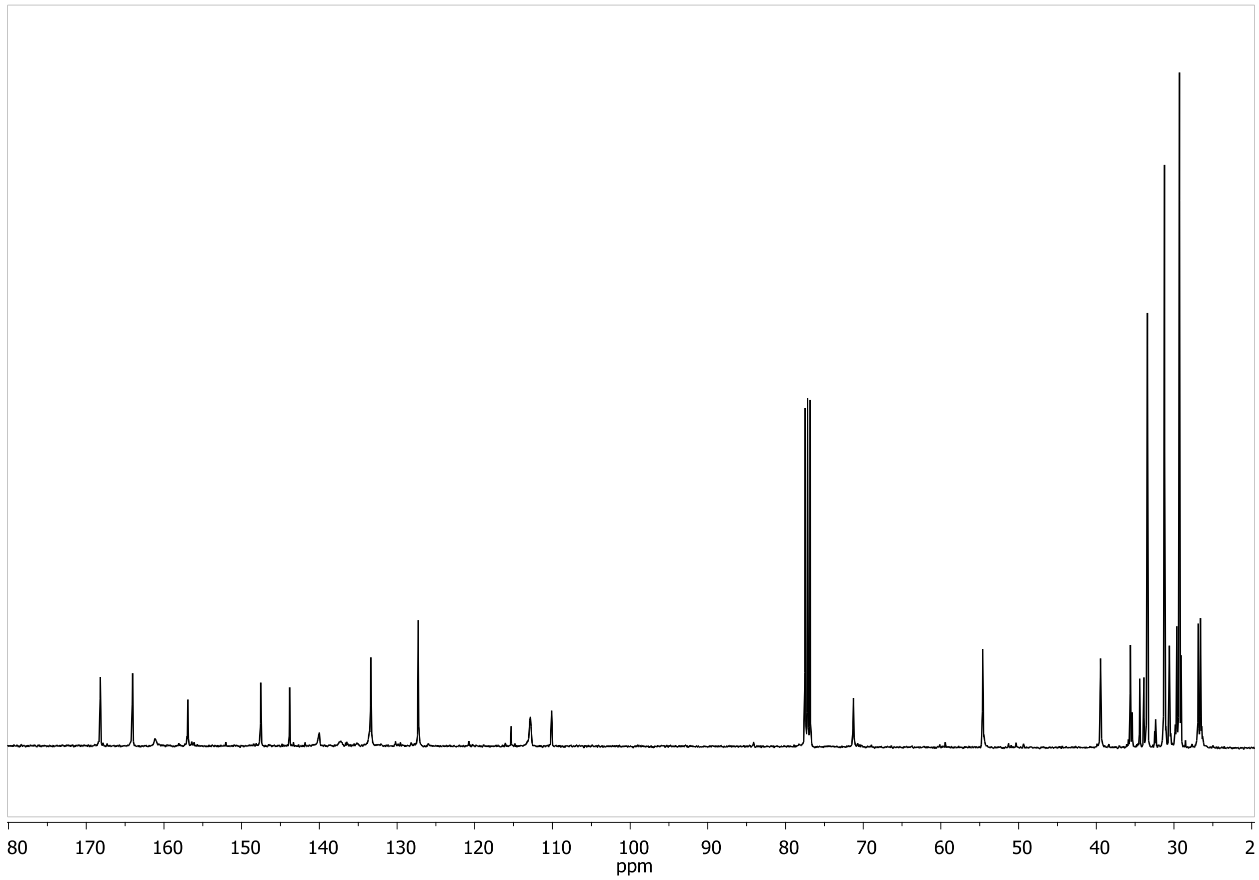

Figure S7. ^{13}C NMR spectrum of the compound **10** (*1,3-alternate*), $CDCl_3$, 298 K, 100 MHz.

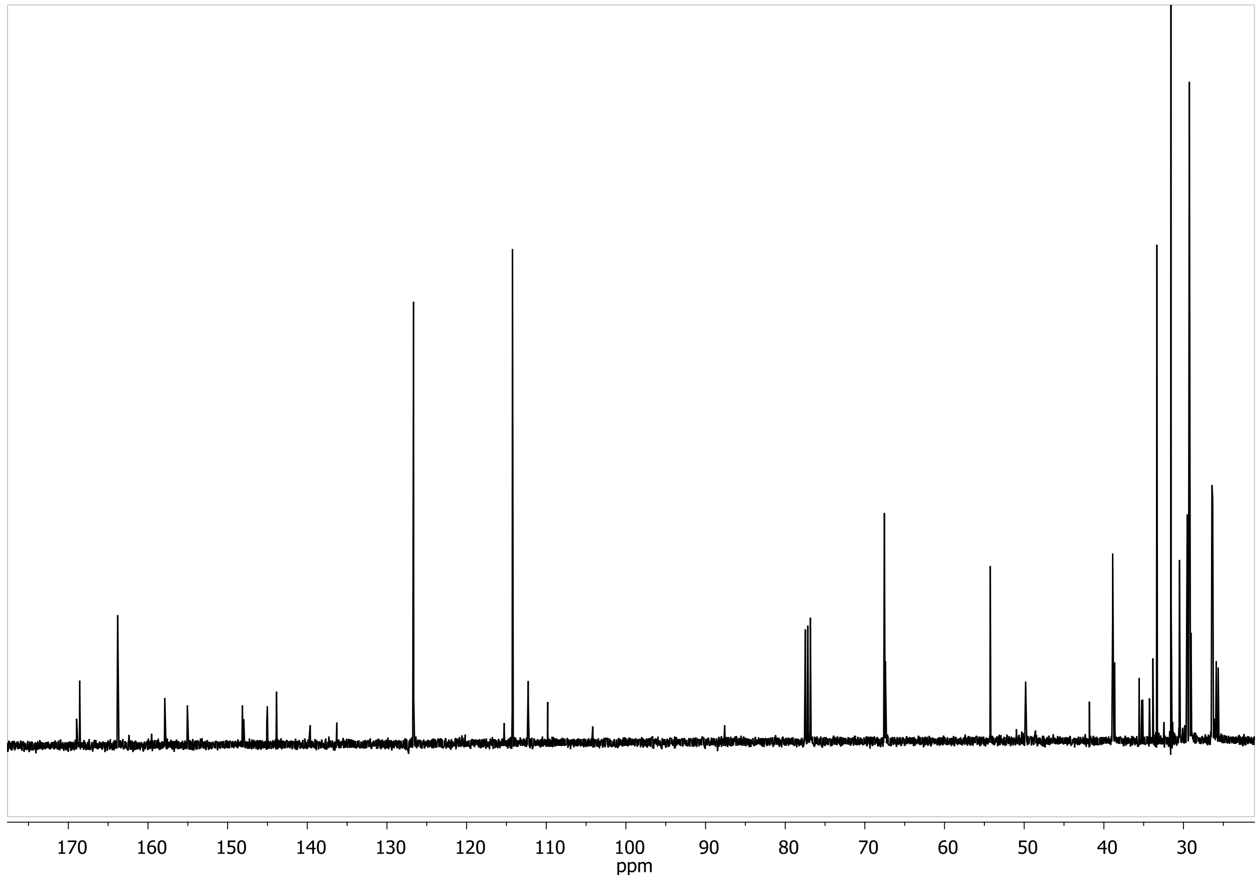

Figure S8. ^{13}C NMR spectrum of the compound **11** (*monomer*), $CDCl_3$, 298 K, 100 MHz.

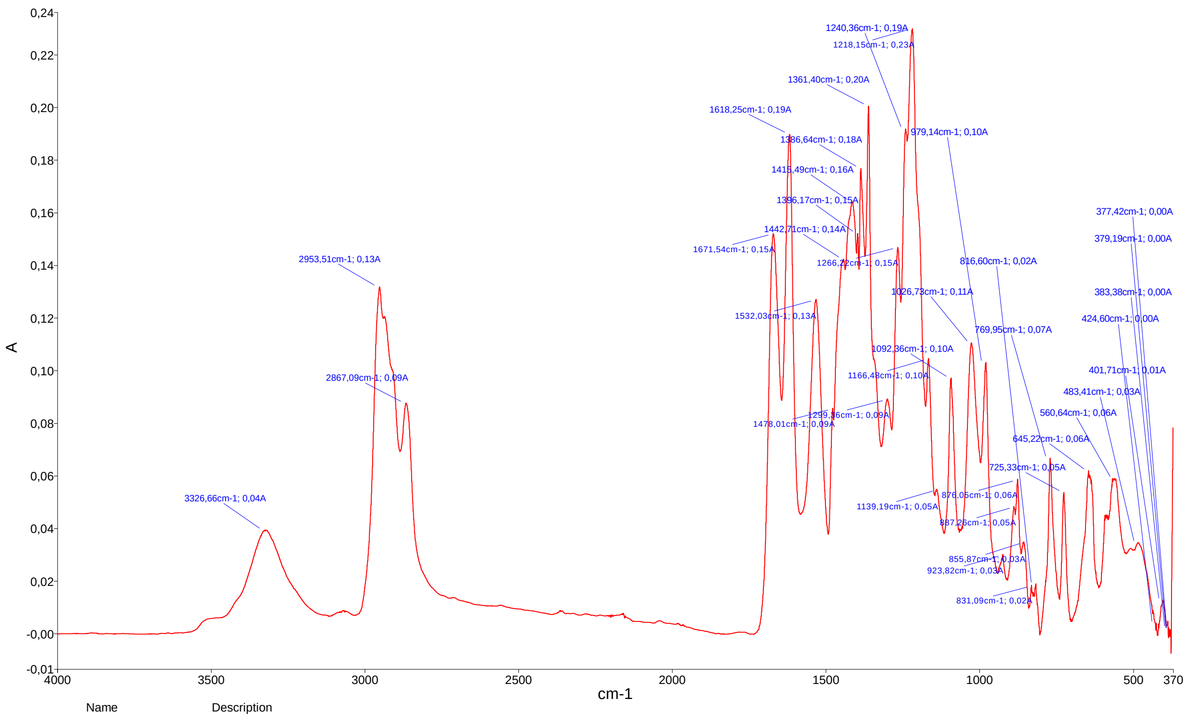

Figure S9. FT-IR spectrum of the compound **8**.

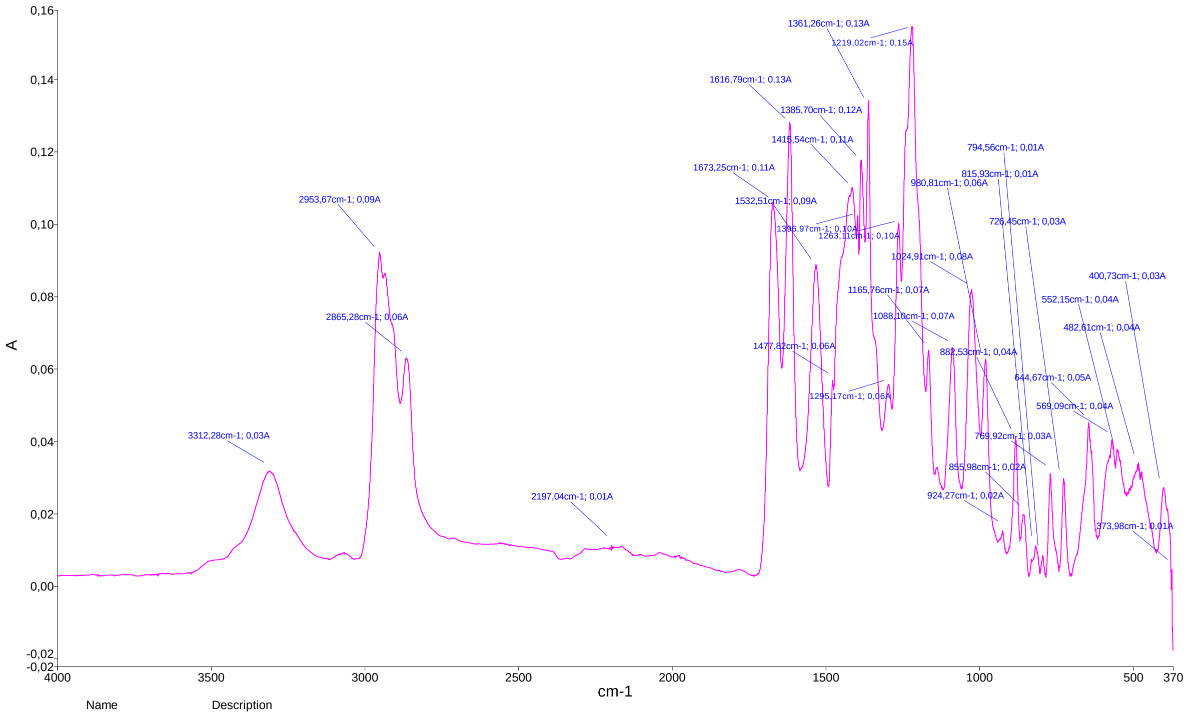

Figure S10. FT-IR spectrum of the compound **9**.

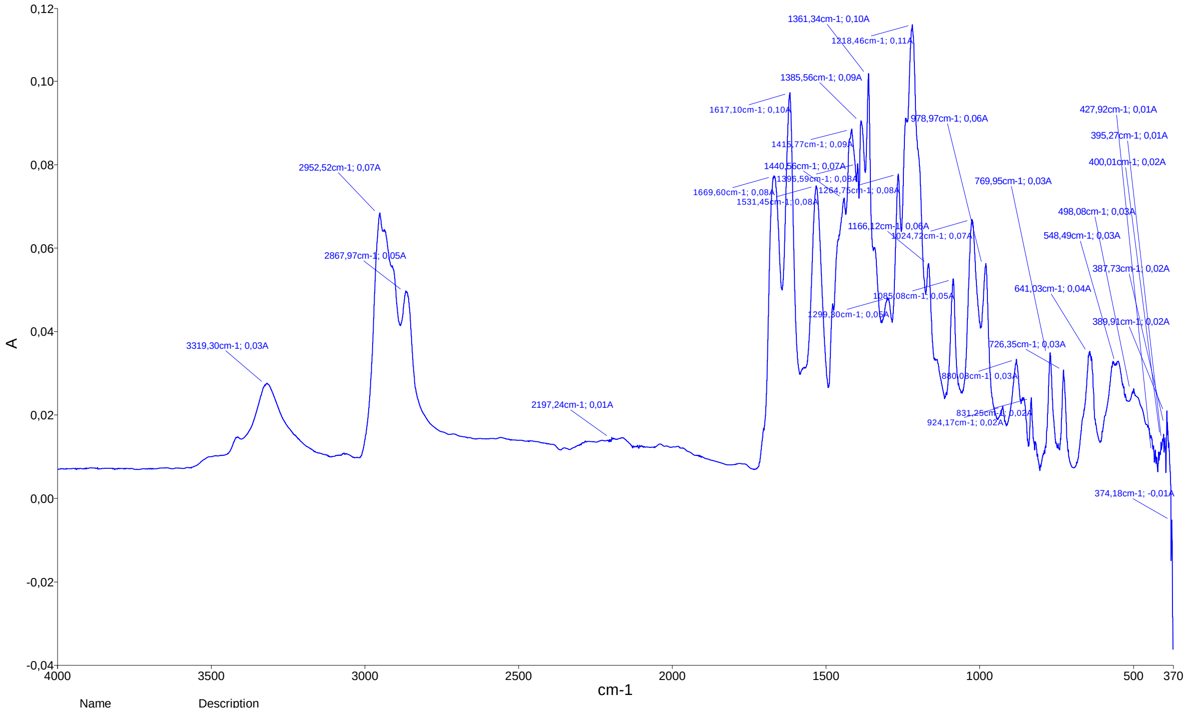

Figure S11. FT-IR spectrum of the compound **10**.

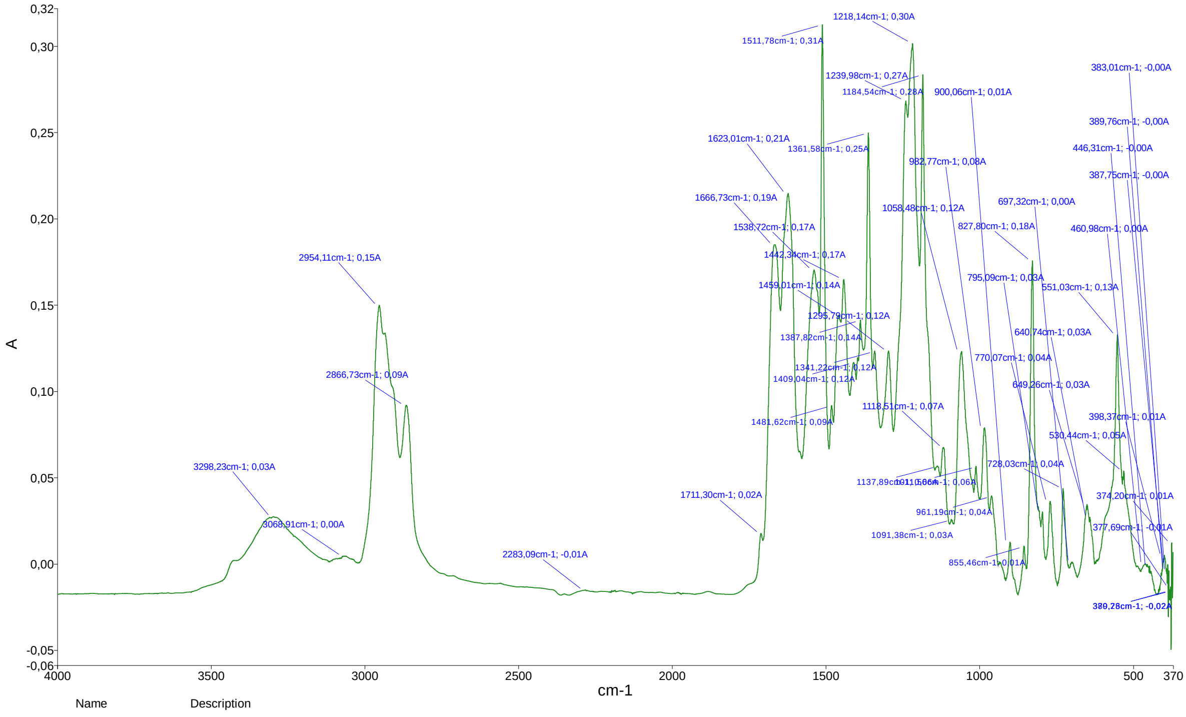

Figure S12. FT-IR spectrum of the compound **11**.

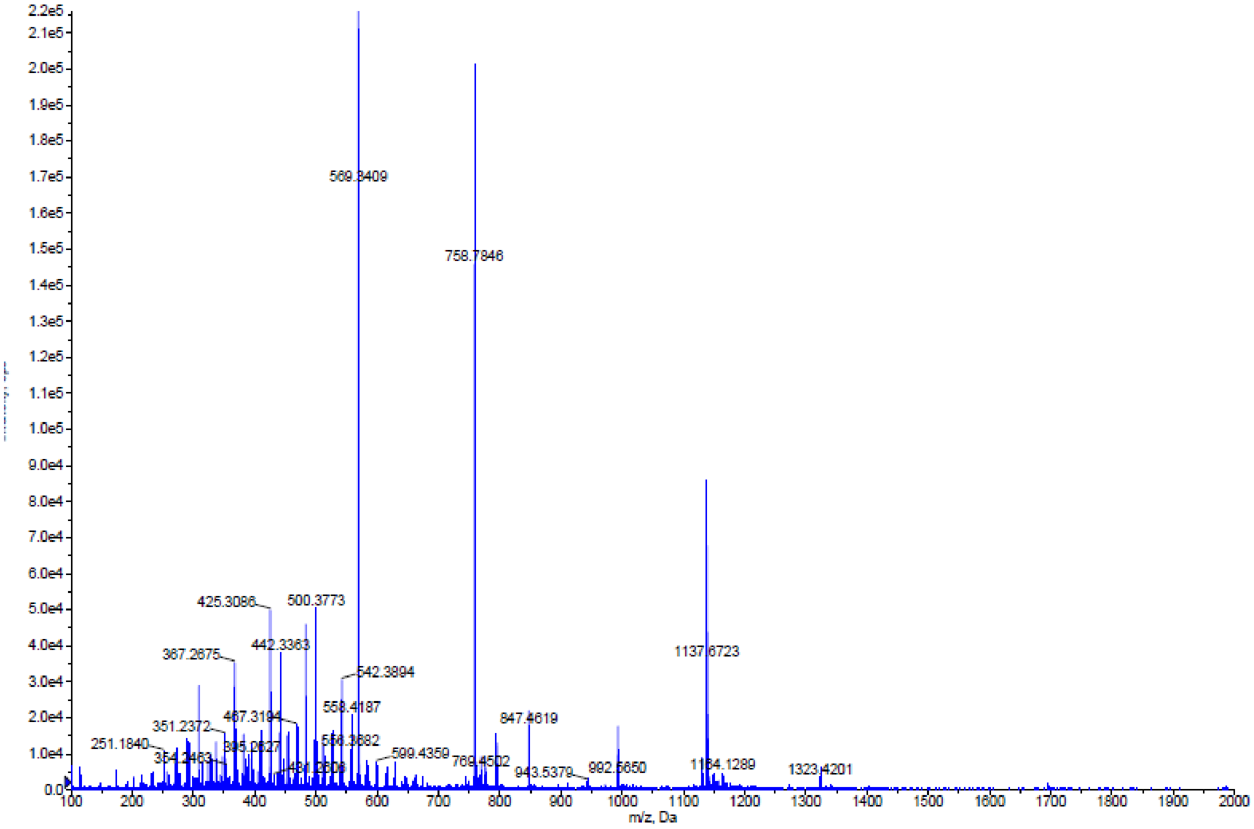

Figure S13. HRMS spectrum of the compound **8**.

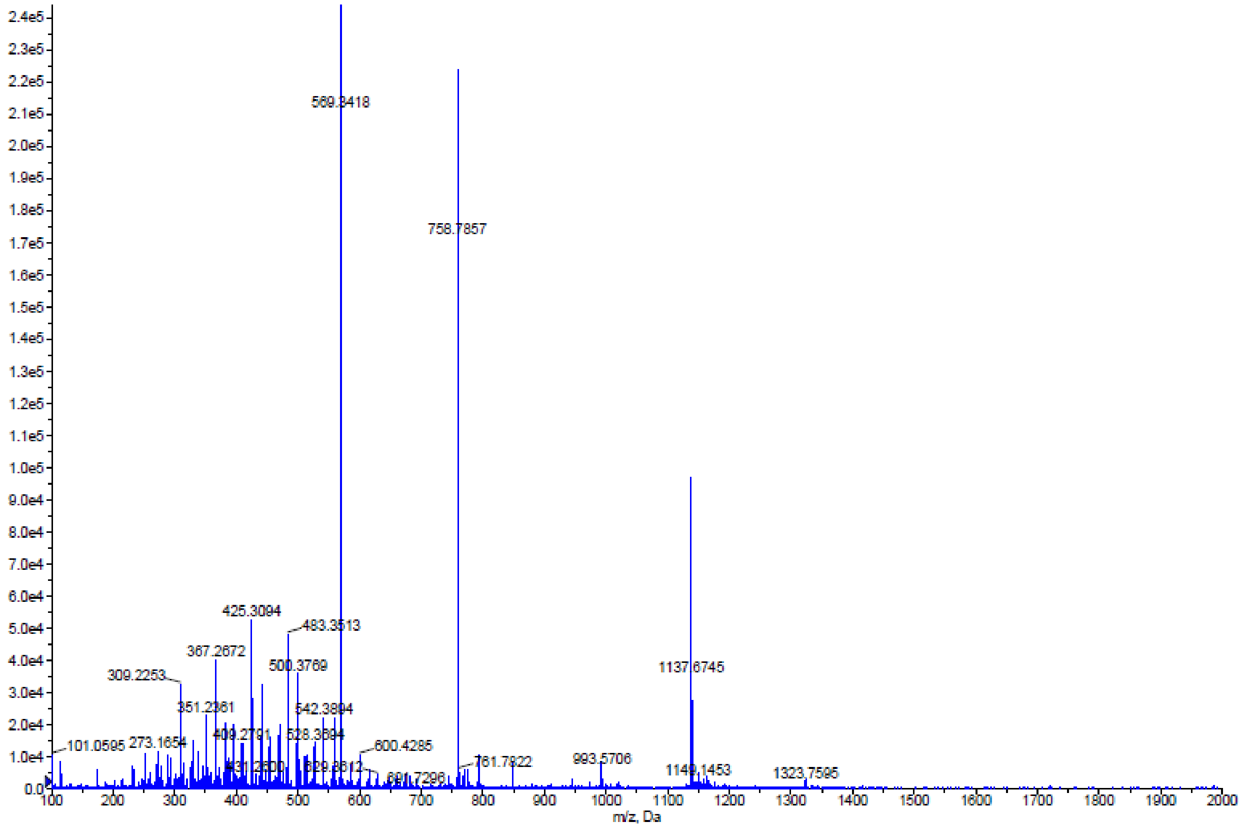

Figure S14. HRMS spectrum of the compound **9**.

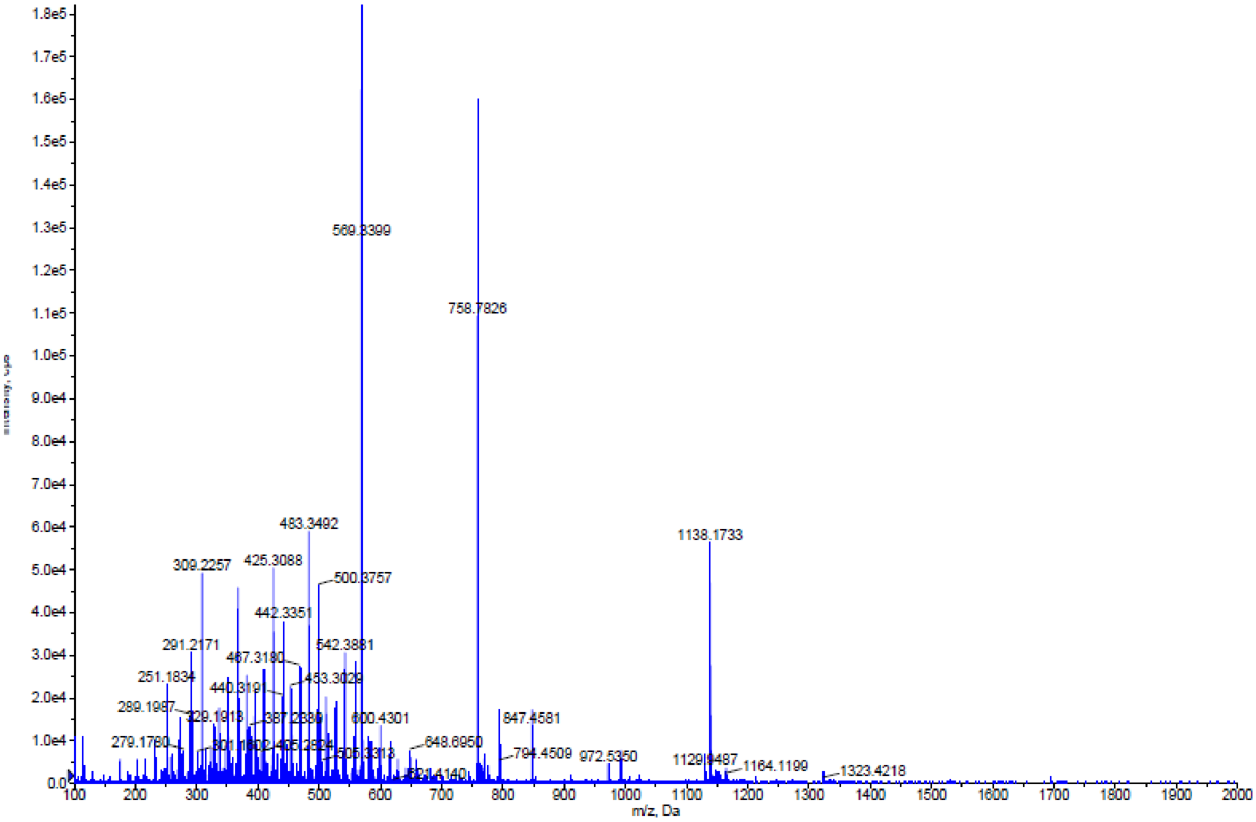

Figure S15. HRMS spectrum of the compound **10**.

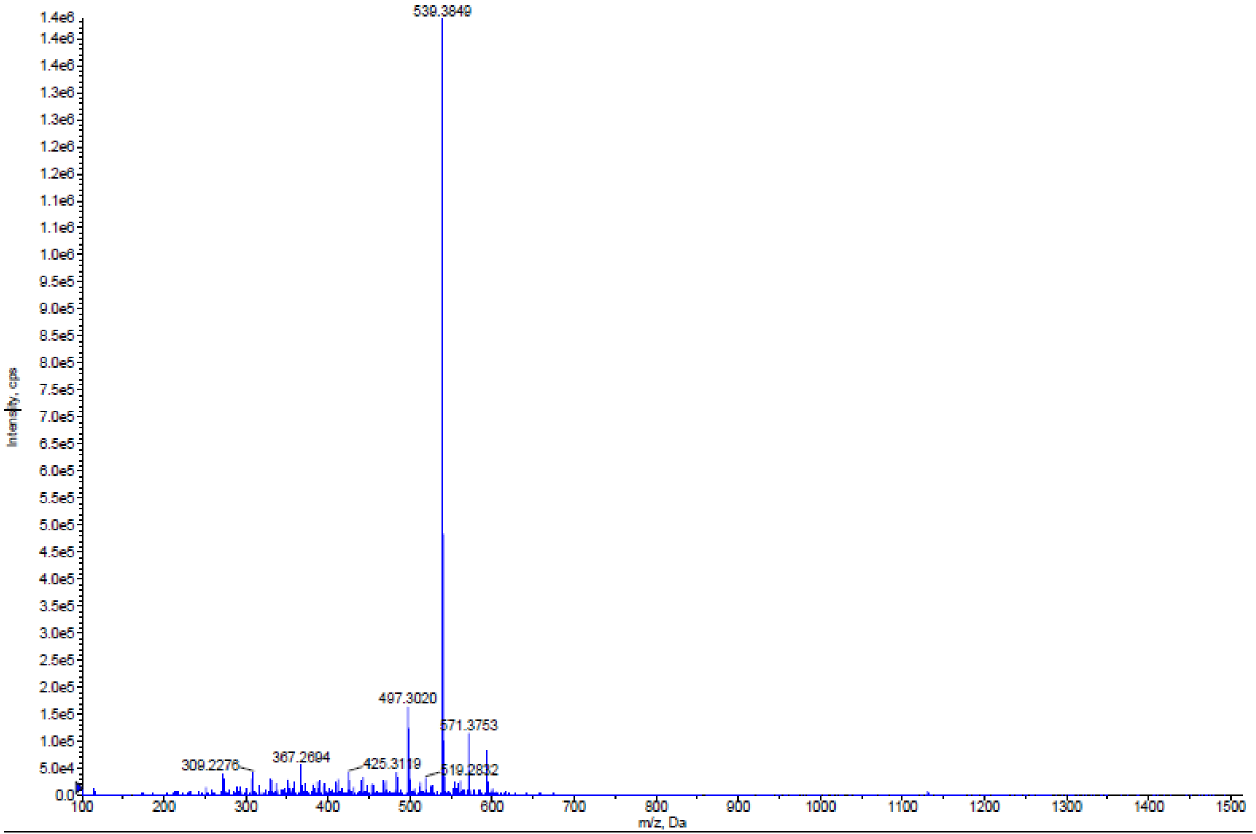

Figure S16. HRMS spectrum of the compound **11**.

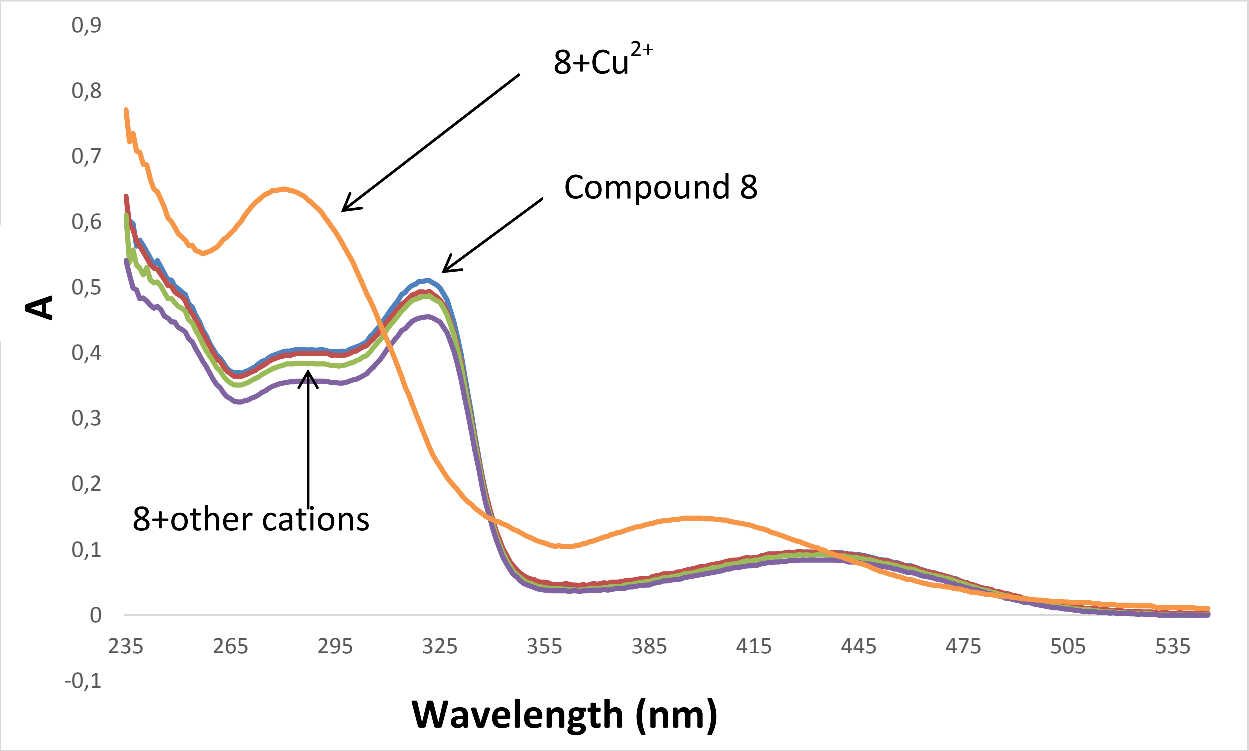

Figure S17. UV-vis spectra of the compound **8** (1×10^{-5} M) without/with 10-fold excess of metal (II) cations (CH_3OH-$CHCl_3$ (1:1), t = 25 °C).

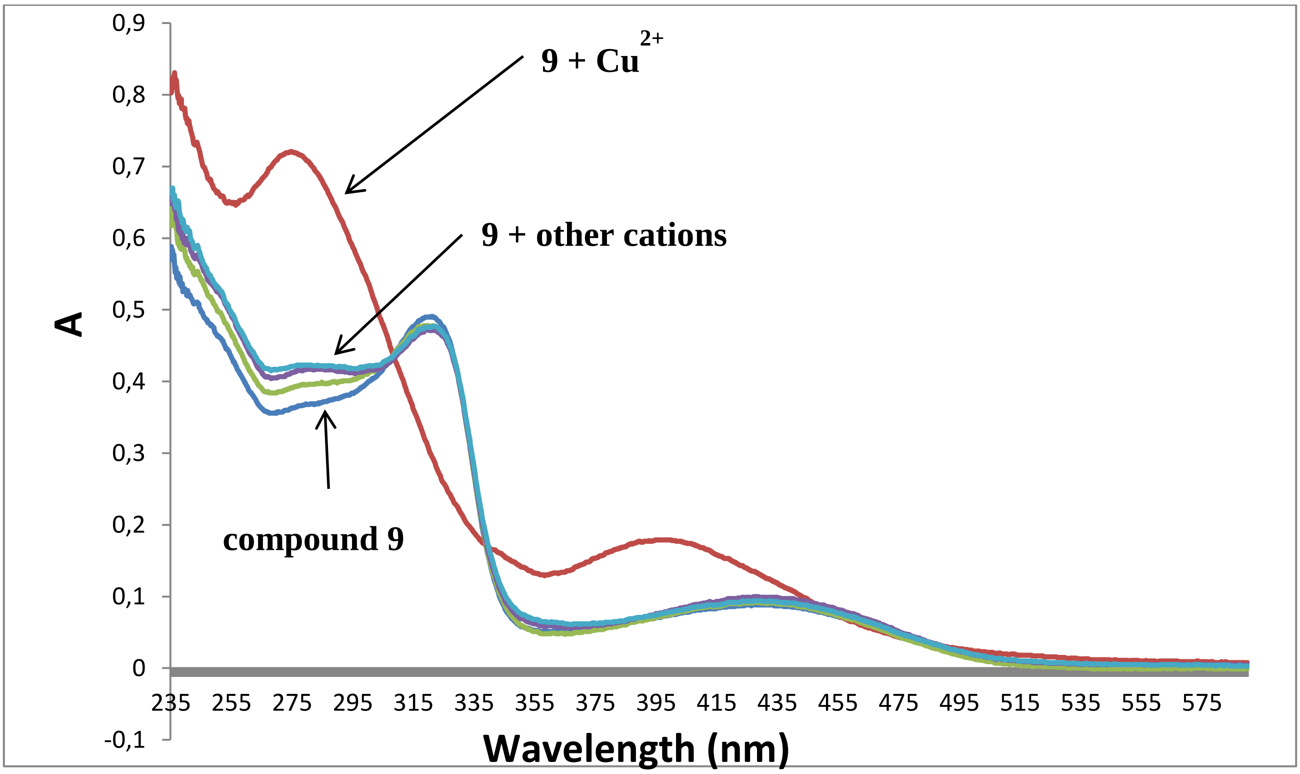

Figure S18. UV-vis spectra of the compound **9** (1×10^{-5} M) without/with 10-fold excess of metal (II) cations (CH_3OH-$CHCl_3$ (1:1), t = 25 °C).

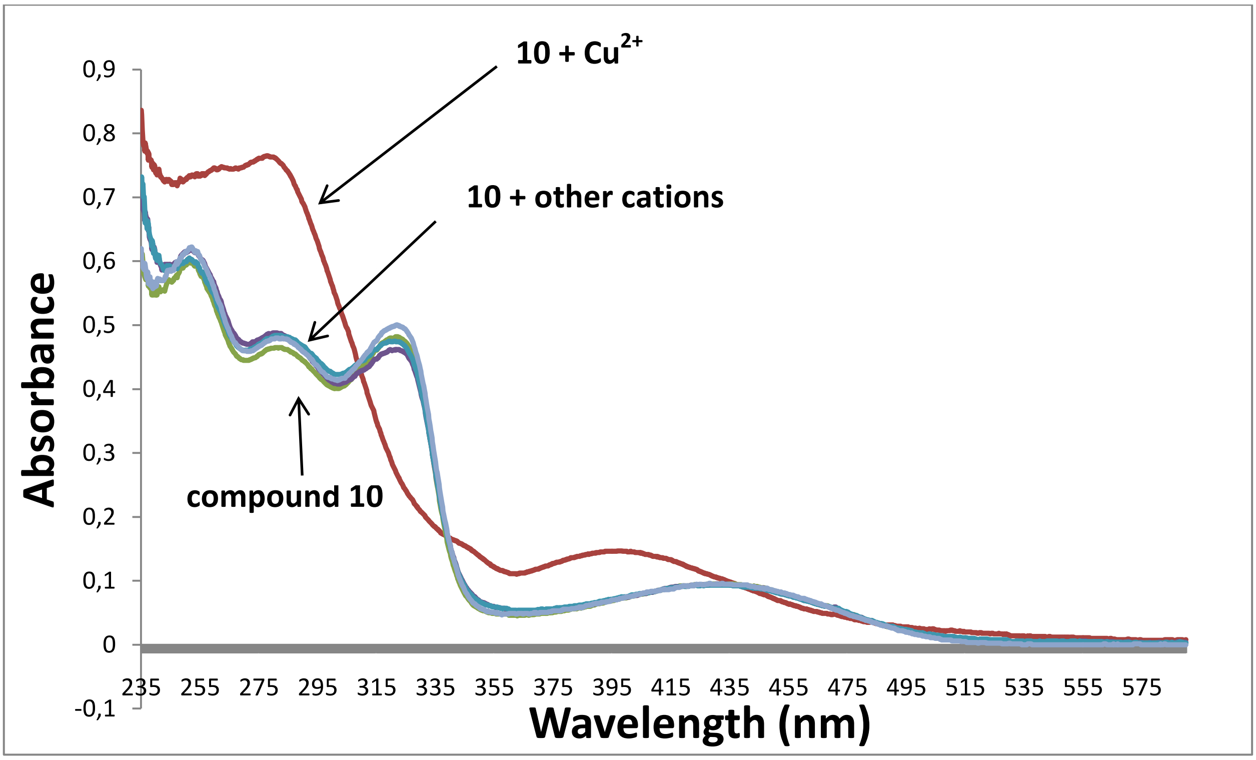

Figure S19. UV-vis spectra of the compound **10** (1×10^{-5} M) without/with 10-fold excess of metal (II) cations (CH_3OH-$CHCl_3$ (1:1), t = 25 °C).

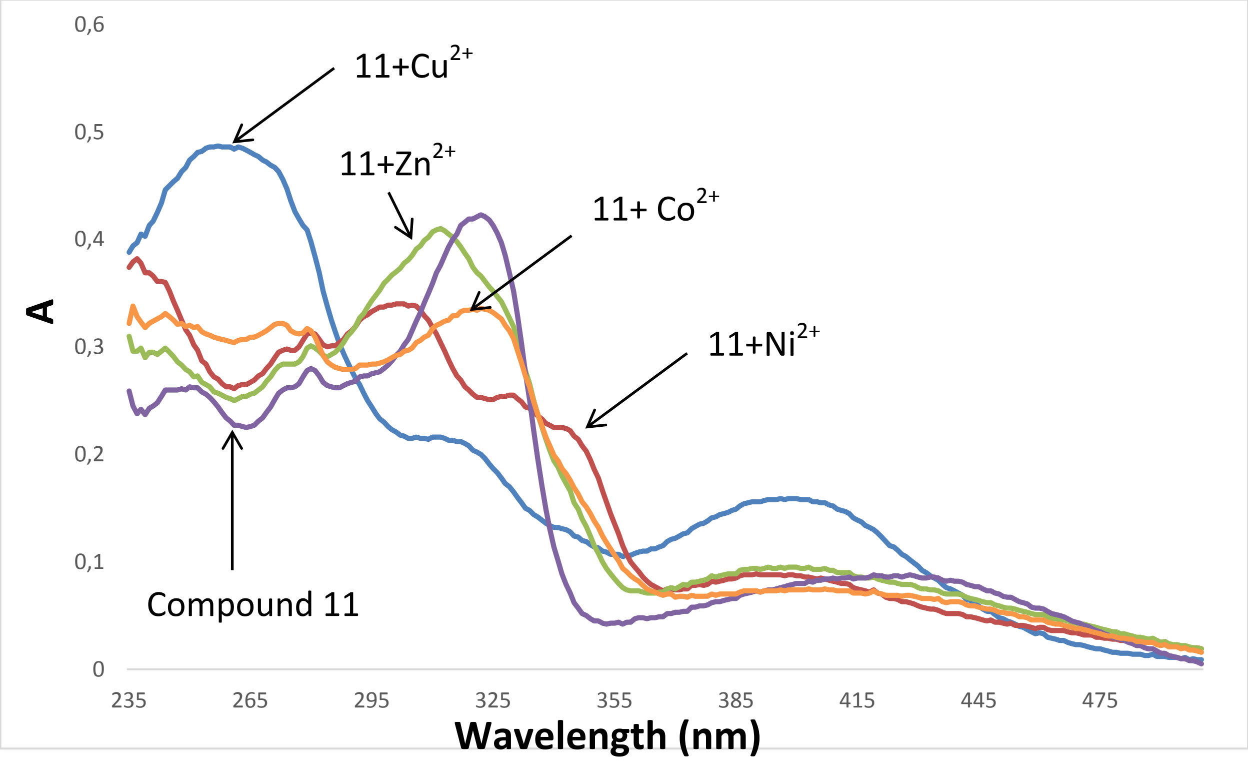

Figure S20. UV-vis spectra of the compound **11** (3×10^{-5} M) without/with 10-fold excess of metal (II) cations (blue 11+Cu^{2+} , green 11+Zn^{2+} , orange 11+Co^{2+} , red 11+Ni^{2+} , violet 11+Cu^{2+}) (CH_3OH-$CHCl_3$ (1:1), t = 25 °C).

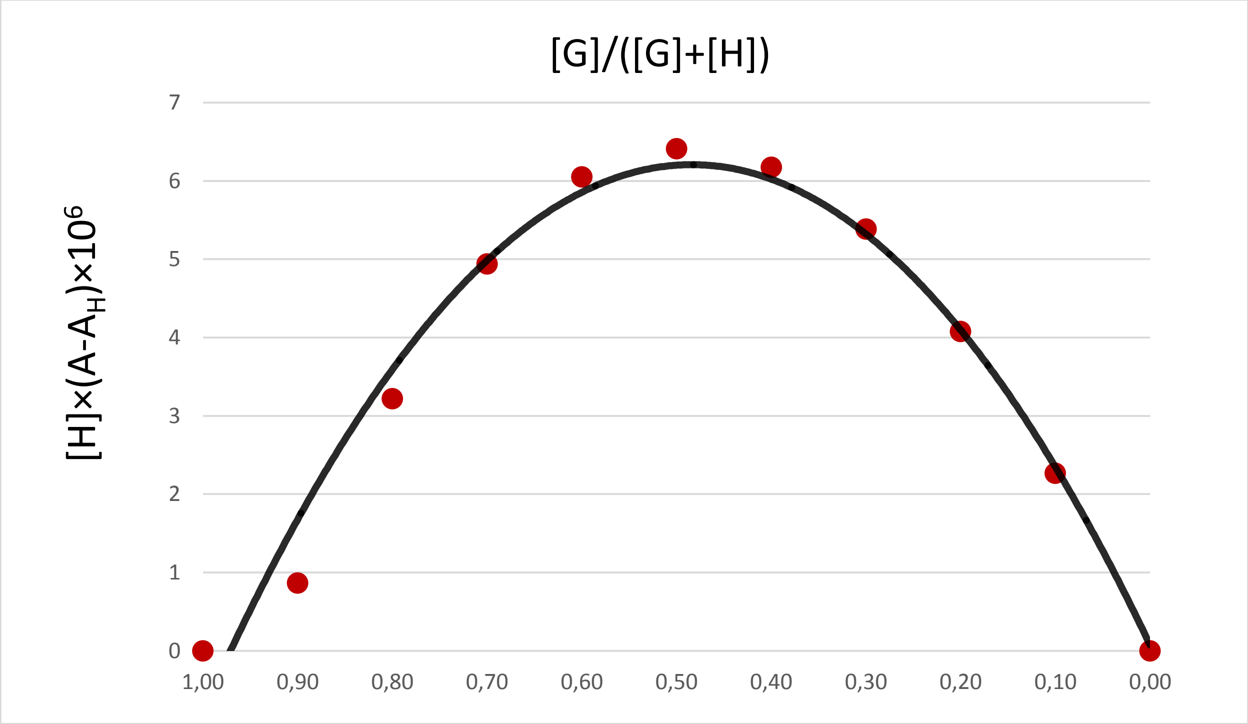

Figure S21. Job's plot for the determination of the stoichiometry in the complex of thiacalix[4]arene **8** (*cone*) and Cu^{2+} in CH_3OH:$CHCl_3$ (1:1).

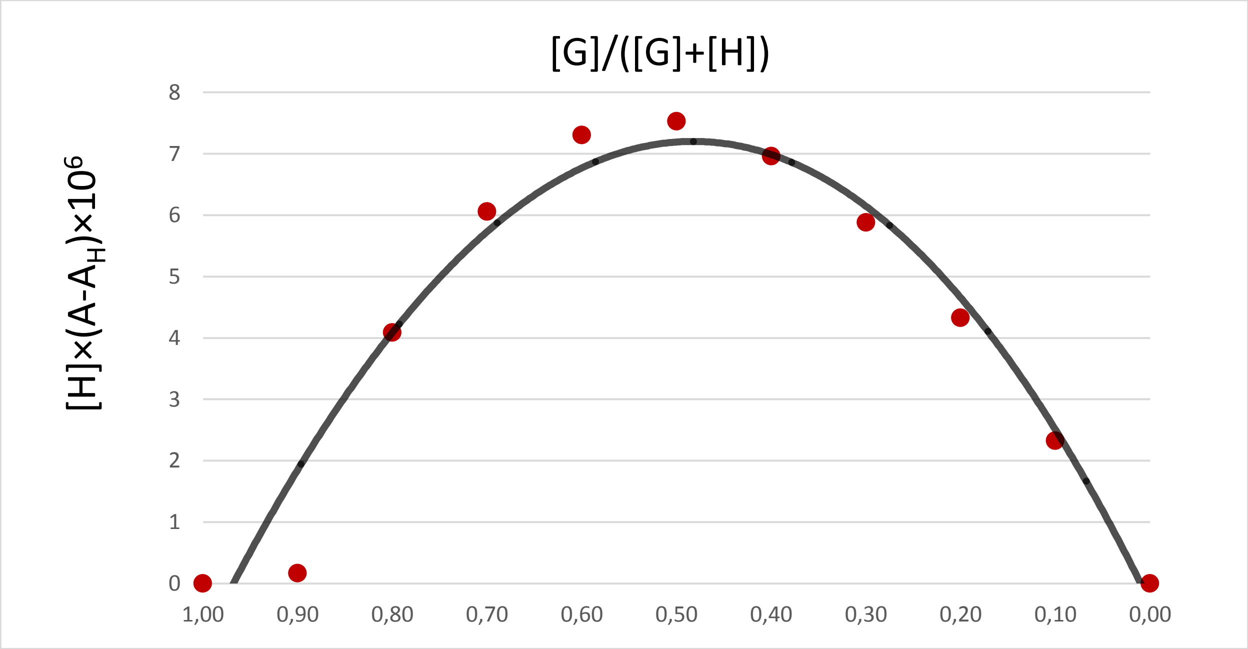

Figure S22. Job's plot for the determination of the stoichiometry in the complex of thiacalix[4]arene **9** (*partial cone*) and Cu^{2+} in CH_3OH:$CHCl_3$ (1:1).

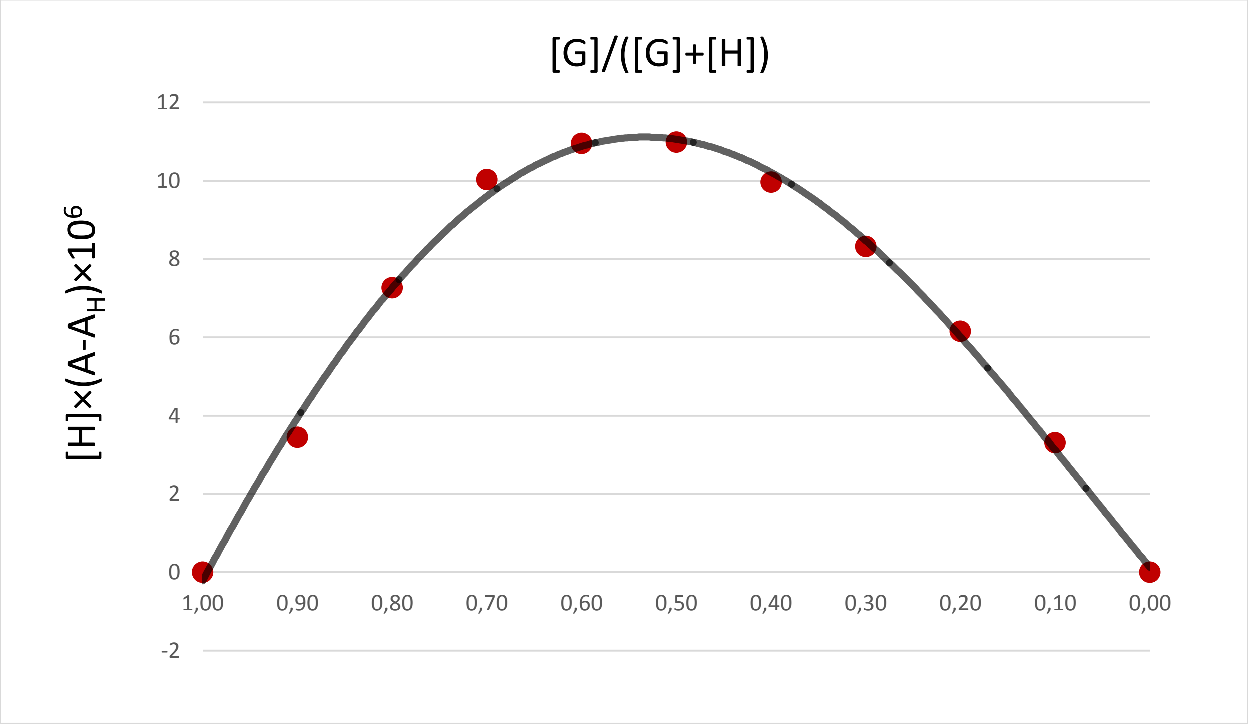

Figure S23. Job's plot for the determination of the stoichiometry in the complex of thiacalix[4]arene **10** (*1,3-alternate*) and Cu^{2+} in $CH_3OH:CHCl_3$ (1:1).

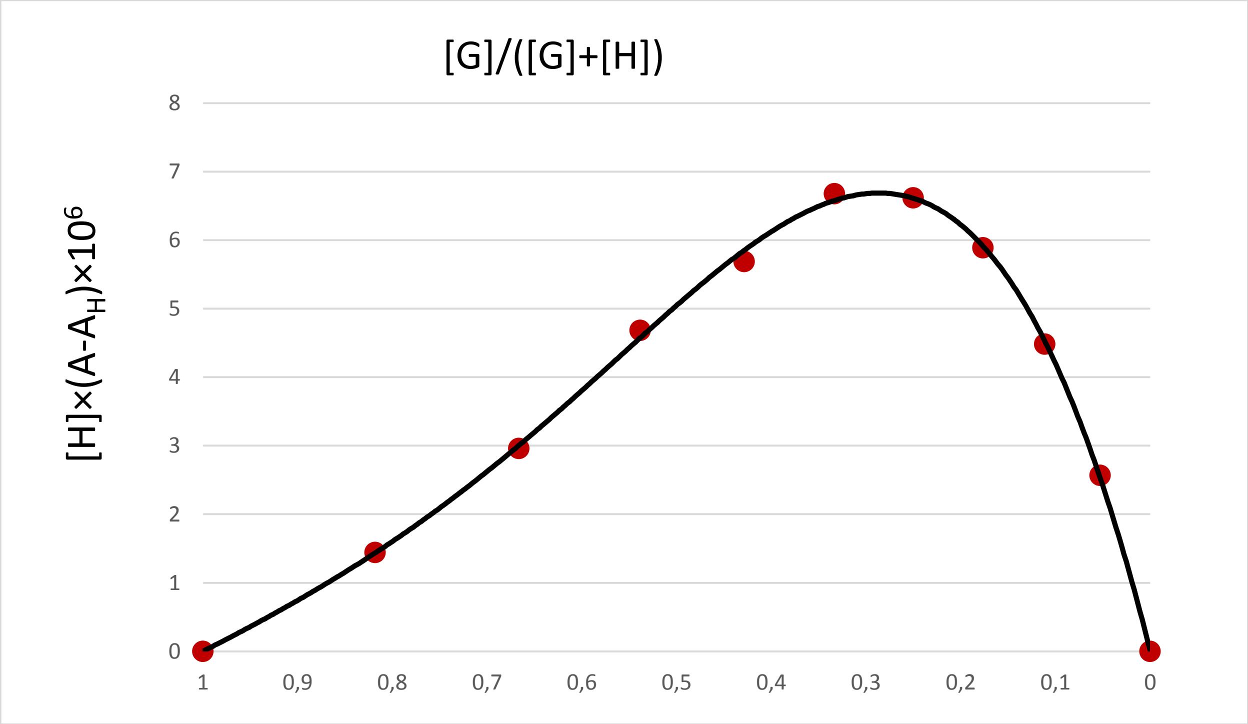

Figure S24. Job's plot for the determination of the stoichiometry in the complex of monomer **11** and Cu^{2+} in $CH_3OH:CHCl_3$ (1:1).

A)

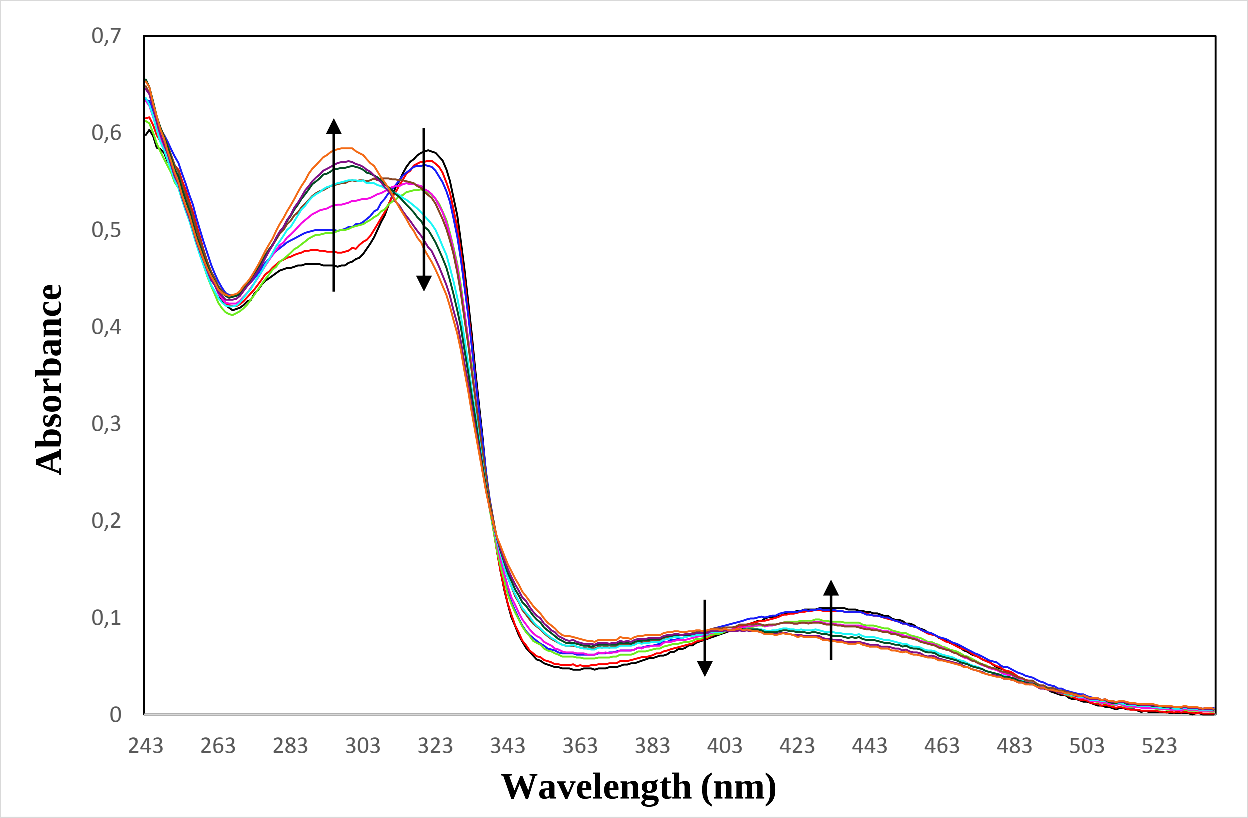

B)

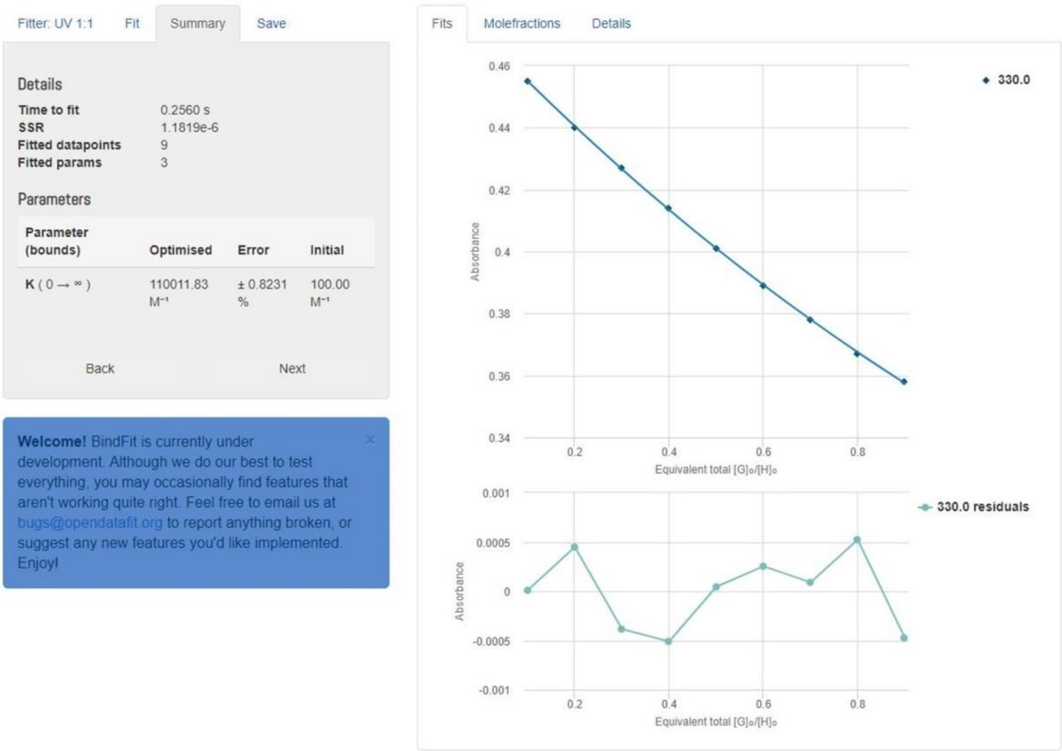

Figure S25. A) UV-Vis spectra of mixtures of thiacalix[4]arene **8** (*cone*) (1×10^{-5} M) with different concentrations of Cu^{2+} (from 0 to 1 equiv) in $CH_3OH:CHCl_3$ (1:1). B) Bindfit (Fit data to 1:1 Host-Guest equilibria). Screenshots taken from the summary window of the website supramolecular.org. This screenshots shows the raw data for UV-Vis titration of the compound 8 with Cu^{2+}.

A)

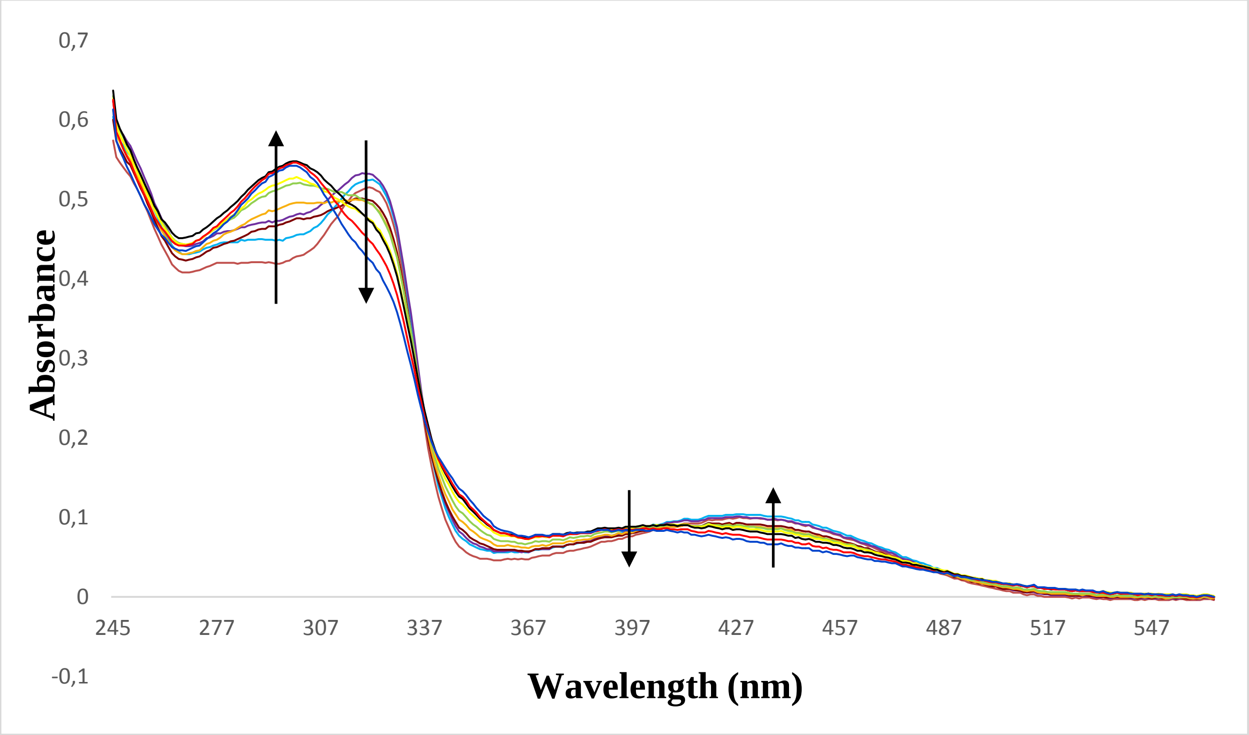

B)

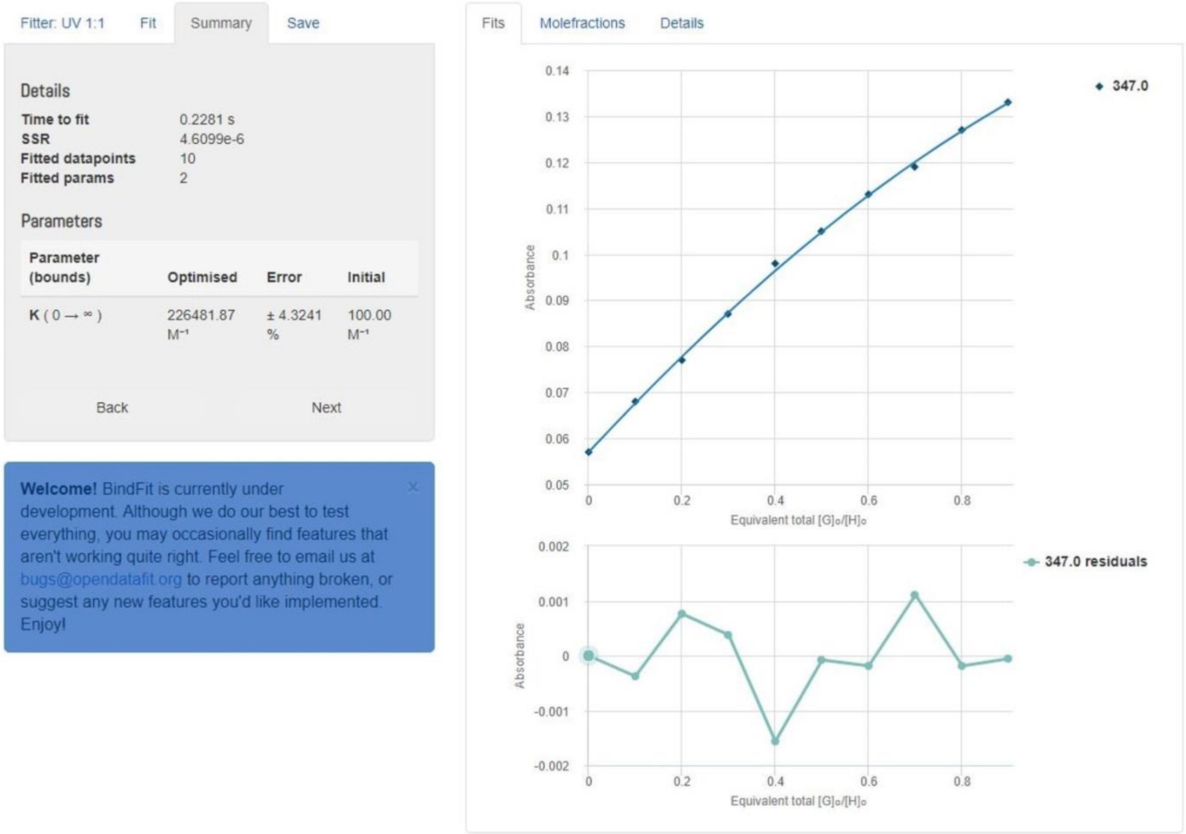

Figure S26. A) UV-Vis spectra of mixtures of thiacalix[4]arene **9** (*partial cone*) (1×10^{-5} M) with different concentrations of Cu^{2+}(from 0 to 1 equiv) in CH_3OH:$CHCl_3$ (1:1). B) Bindfit (Fit data to 1:1 Host-Guest equilibria). Screenshots taken from the summary window of the website supramolecular.org. This screenshots shows the raw data for UV-Vis titration of the compound 9 with Cu^{2+}.

A)

B)

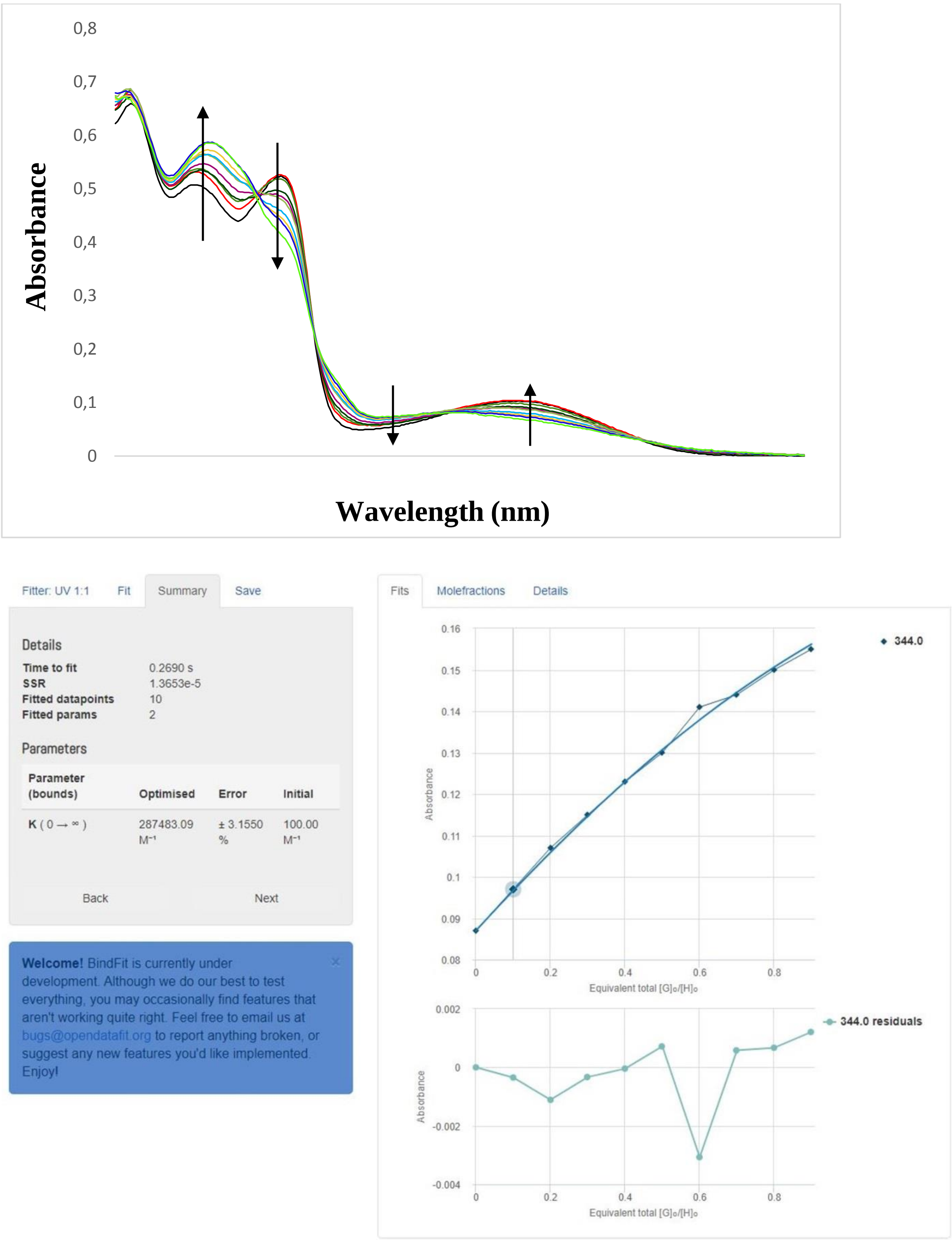

Figure S27. A) UV-Vis spectra of mixtures of thiacalix[4]arene **10** (*1,3-alternate*) (1×10^{-5} M) with different concentrations of Cu^{2+} in $CH_3OH:CHCl_3$ (1:1). B) Bindfit (Fit data to 1:1 Host-Guest equilibria). Screenshots taken from the summary window of the website supramolecular.org. This screenshots shows the raw data for UV-Vis titration of the compound 10 with Cu^{2+}.

A)

B)

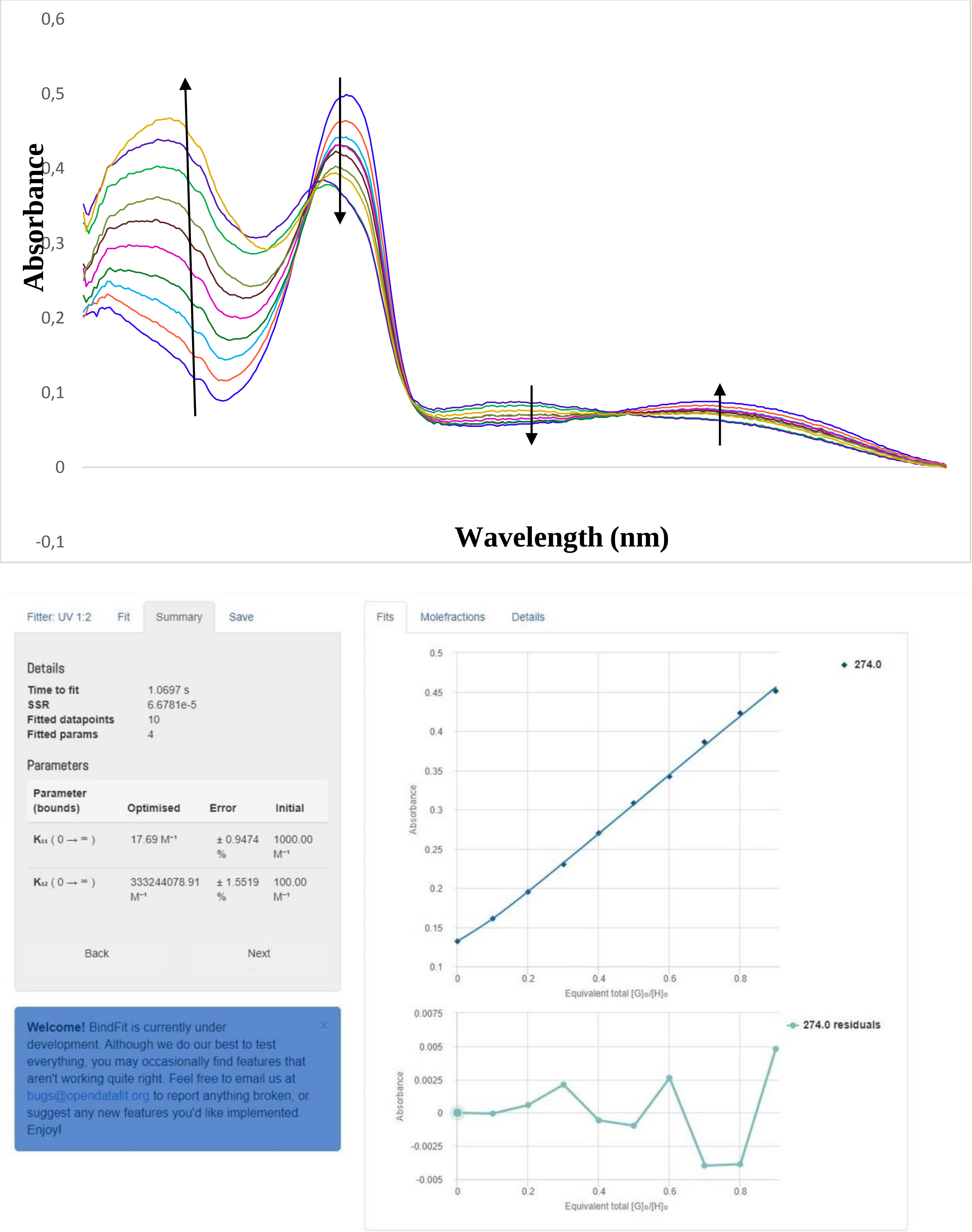

Figure S28. A) UV-Vis spectra of mixtures of monomer **11** (3×10^{-5} M) with different concentrations of Cu^{2+} (from 0 to 1 equiv) in $CH_3OH:CHCl_3$ (1:1). B) Bindfit (Fit data to 2:1 Host-Guest equilibria). Screenshots taken from the summary window of the website supramolecular.org. This screenshots shows the raw data for UV-Vis titration of the compound 11 with Cu^{2+}.

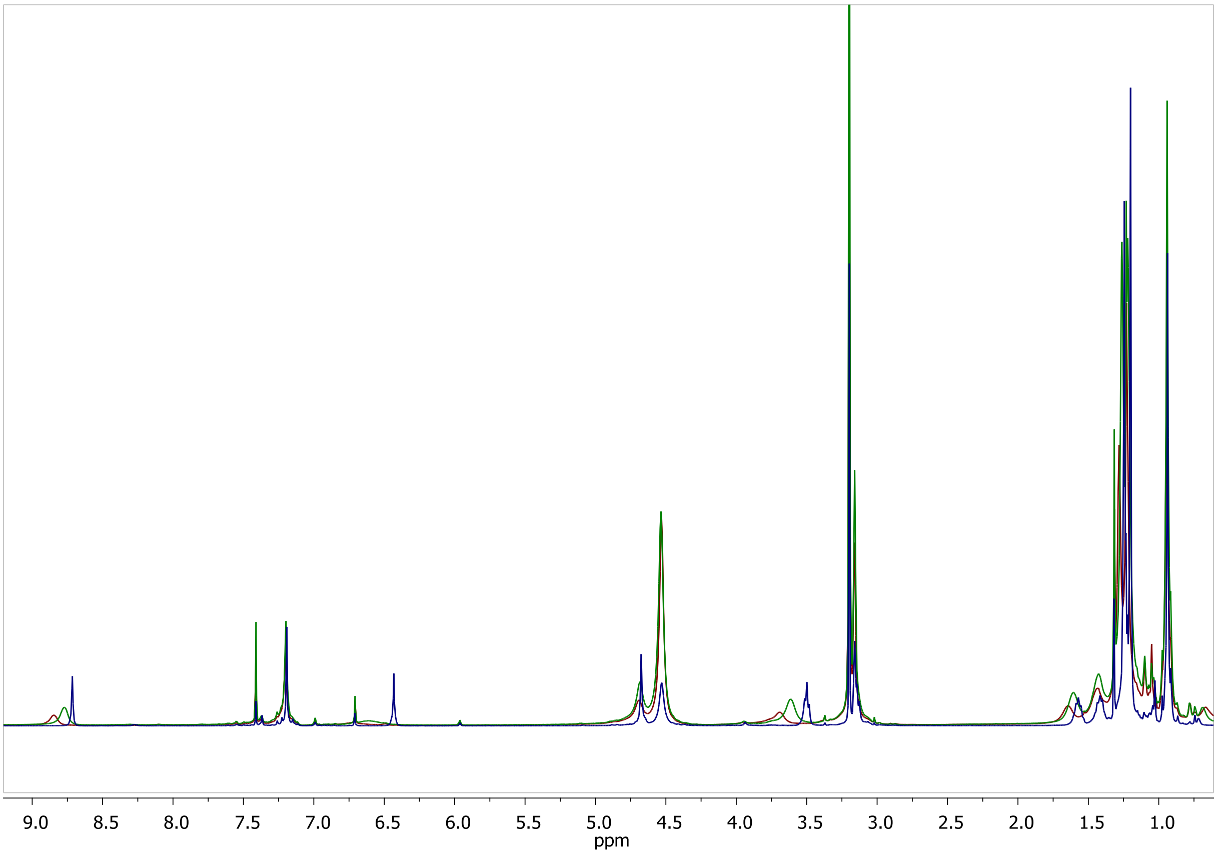

Figure S29. ^{1}H NMR spectra of the compound **8** (10 mM) in $CD_3OD:CDCl_3$ (1:1) upon addition of 0 equiv (blue), 0.5 equiv (green), 1 equiv of Cu^{2+} (red).

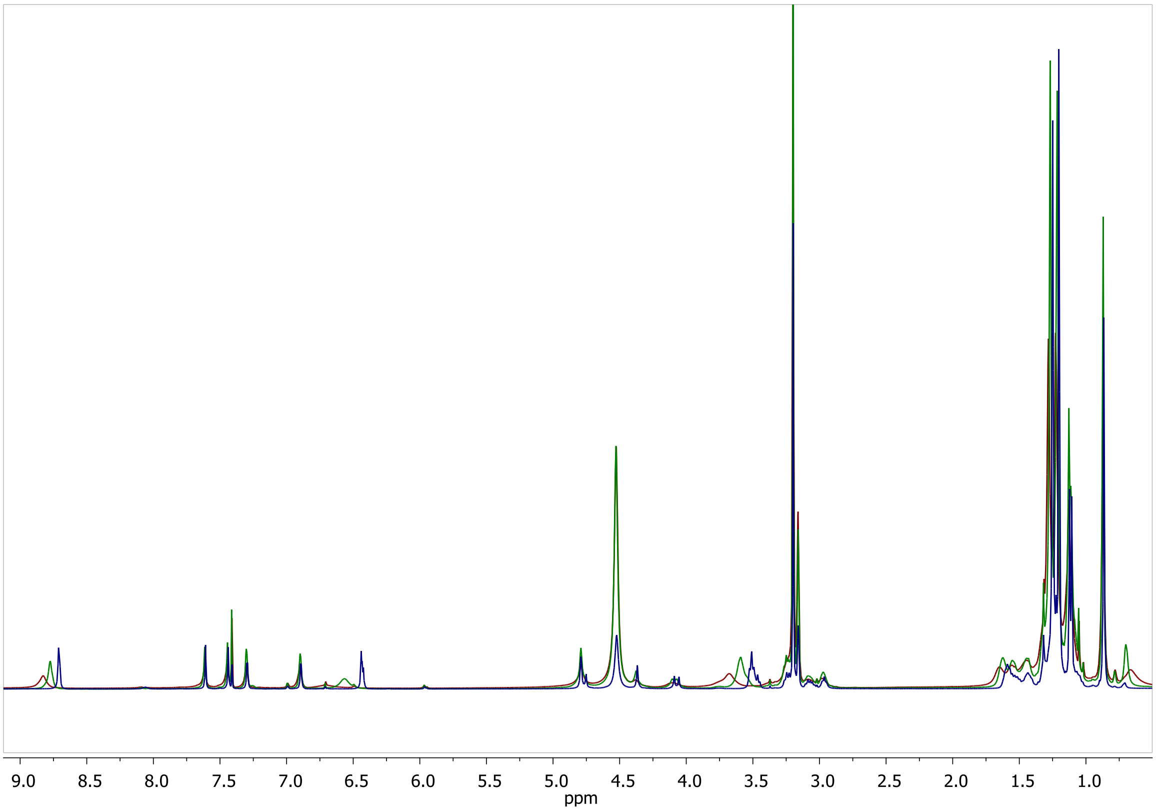

Figure S30. ^{1}H NMR spectra of the compound **9** (10 mM) in $CD_3OD:CDCl_3$ (1:1) upon addition of 0 equiv (blue), 0.5 equiv (green), 1 equiv of Cu^{2+} (red).

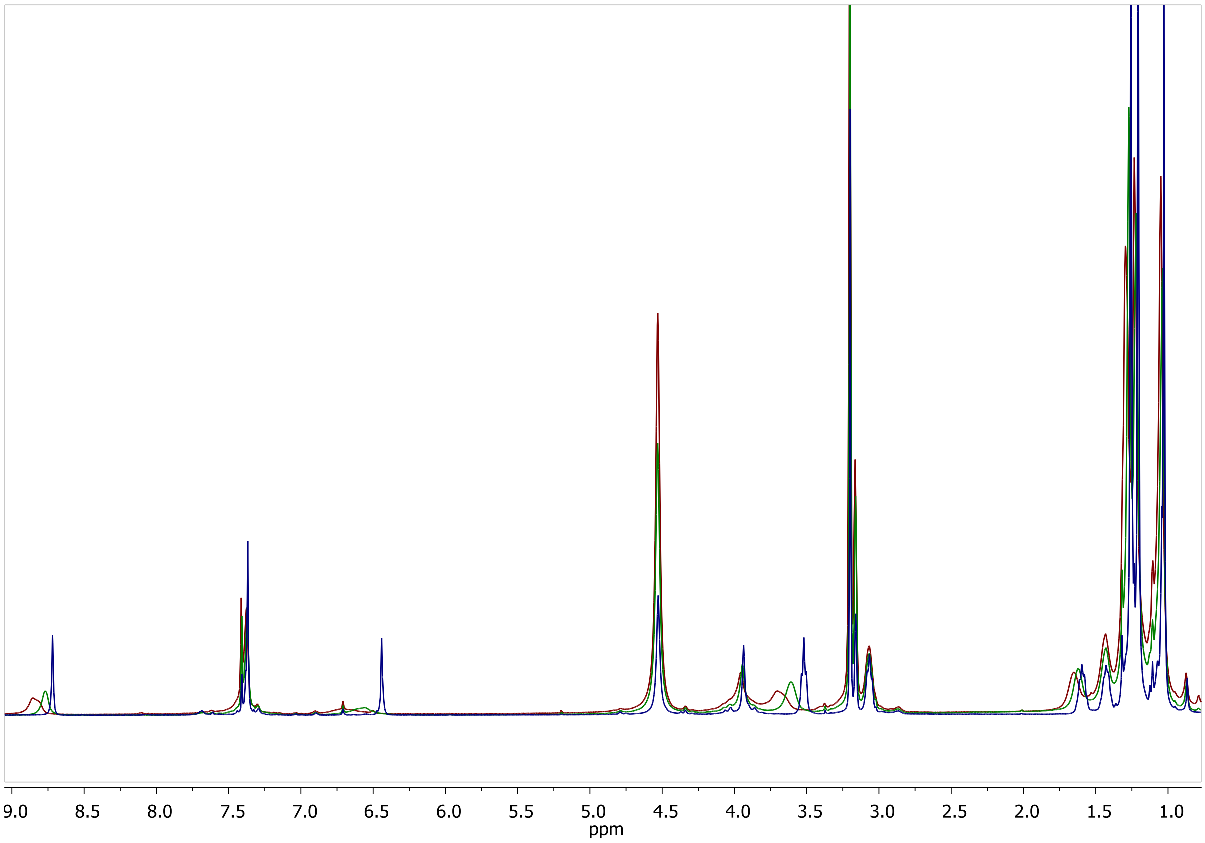

Figure S31. ^{1}H NMR spectra of the compound **10** (10 mM) in $CD_3OD:CDCl_3$ (1:1) upon addition of 0 equiv (blue), 0.5 equiv (green), 1 equiv of Cu^{2+} (red).

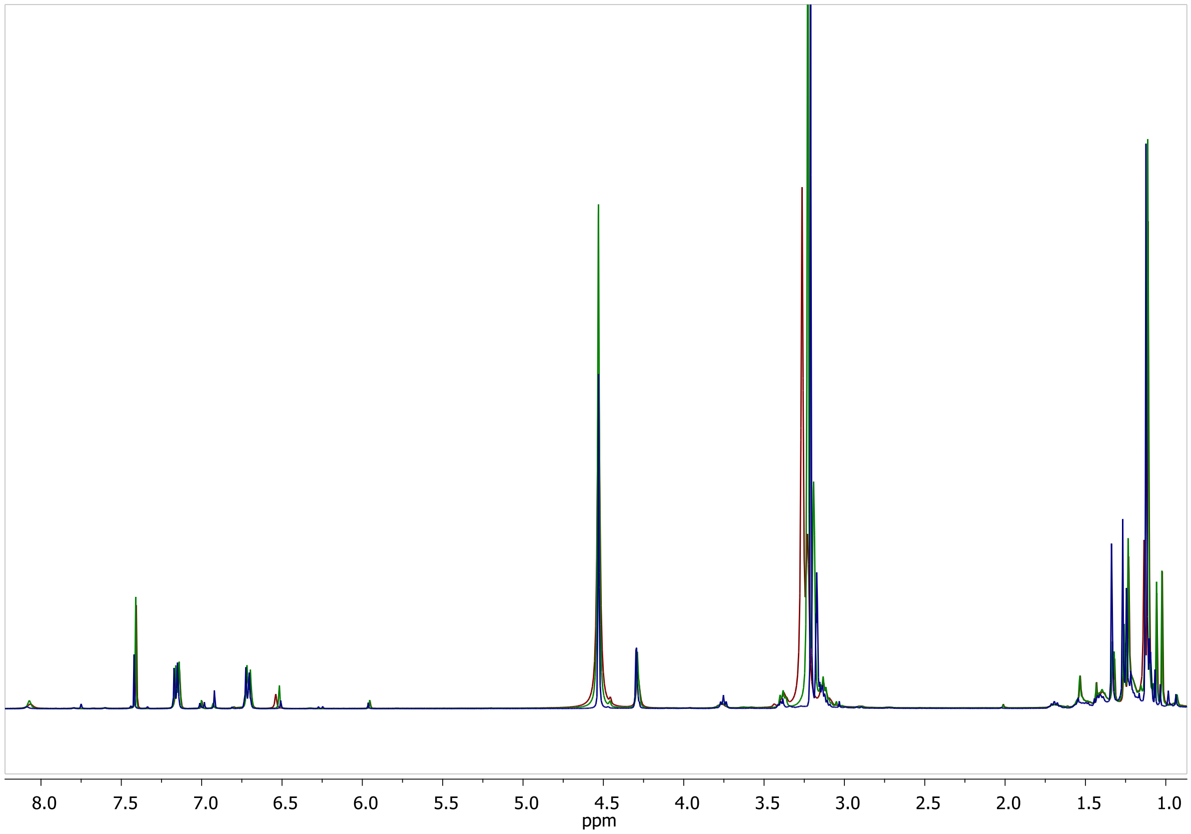

Figure S32. ^{1}H NMR spectra of the compound **11** (10 mM) in $CD_3OD:CDCl_3$ (1:1) upon addition of 0 equiv (blue), 0.5 equiv (green), 1 equiv of Cu^{2+} (red).

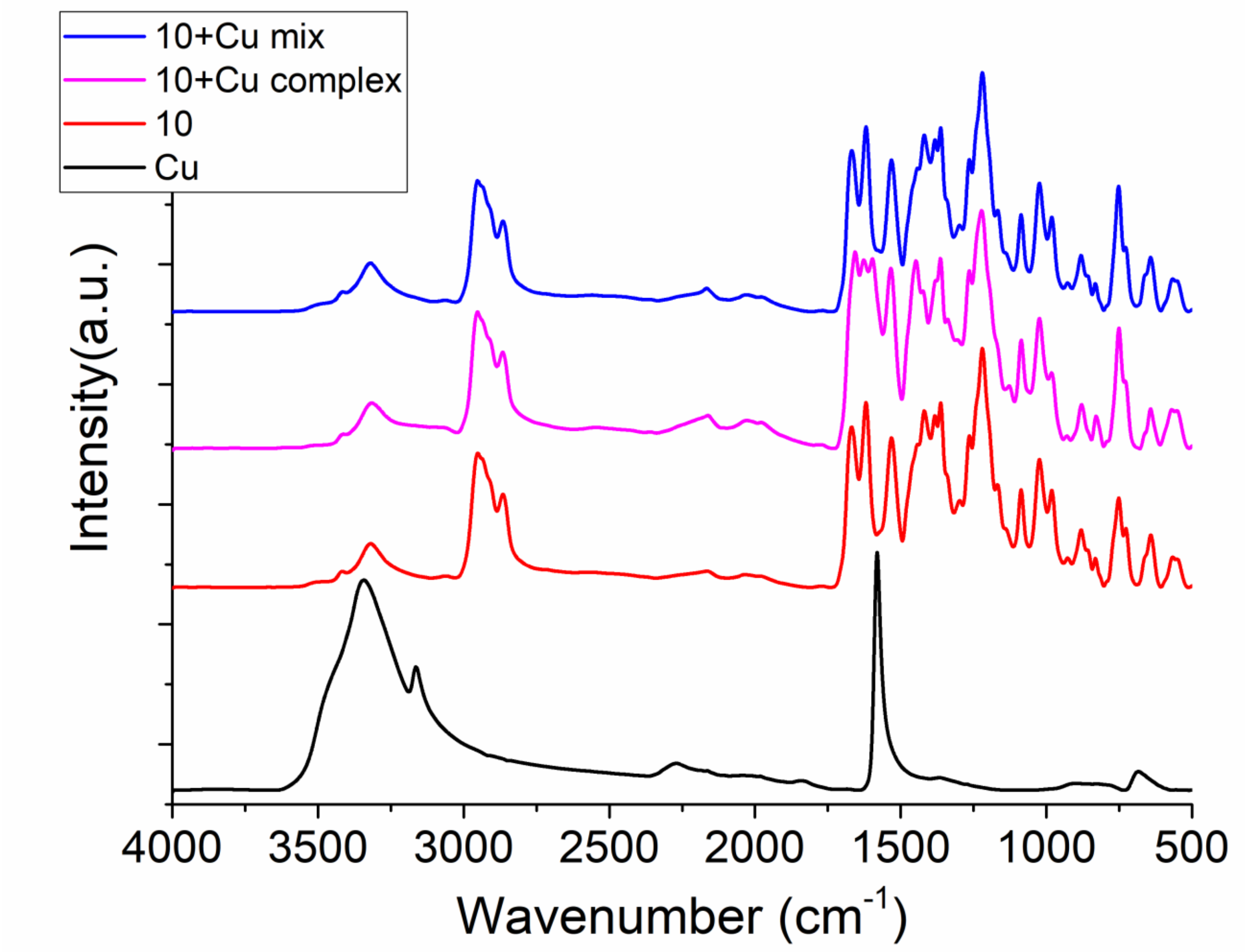

Figure S33. FT-IR spectra of the compound **10** (black), **10**+Cu mixture (red), **10**+Cu complex (blue) and $CuCl_2$ (purple).

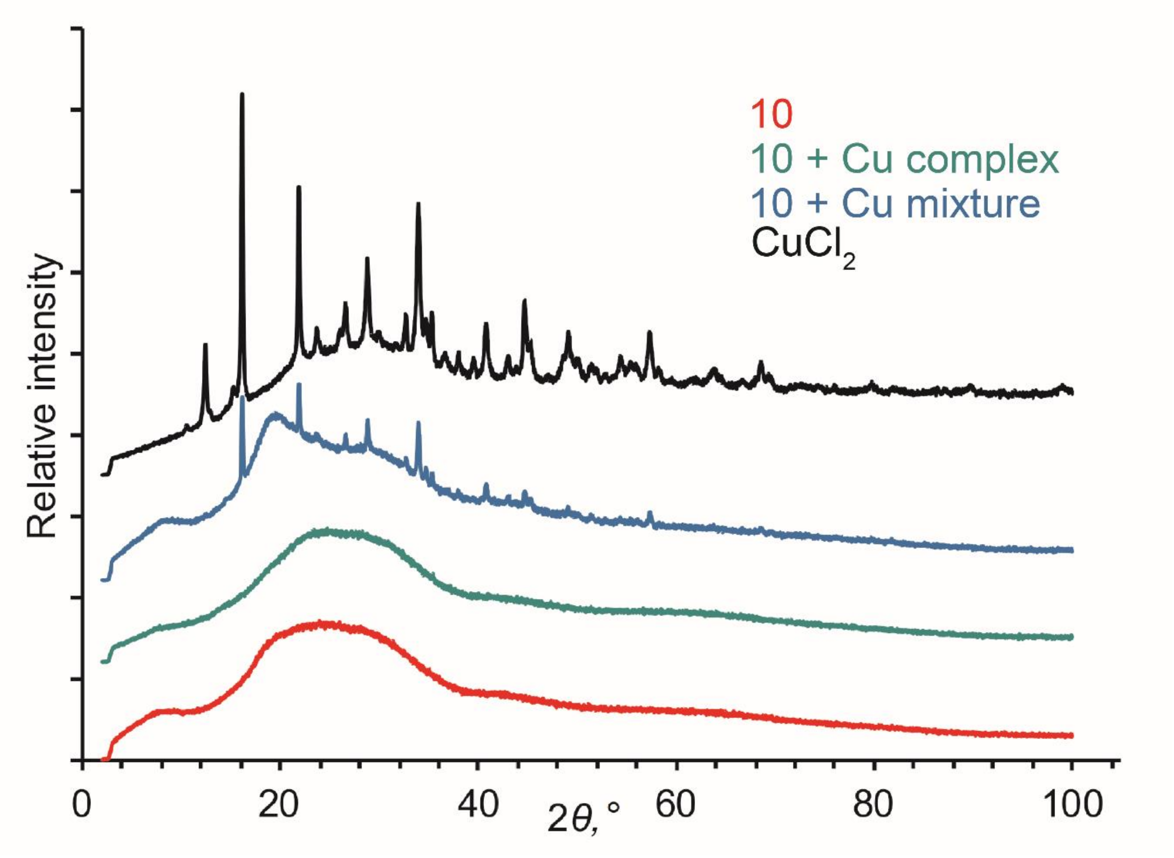

Figure S34. Powder X-ray diffractograms of the compound **10**, $CuCl_2$, **10**+Cu mixture and copper based material (**10**+Cu complex).

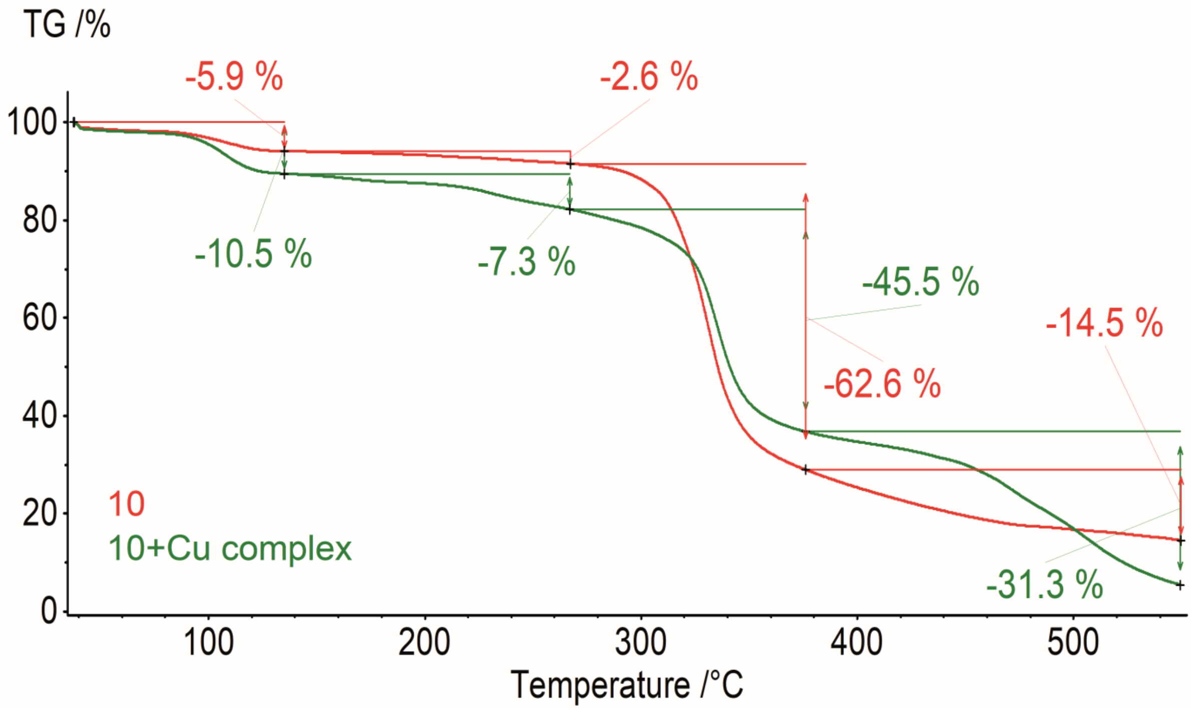

Figure S35. TG curves of the compound **10** (red) and **10**+Cu complex (green) in the dynamic argon atmosphere 75 ml/min in the temperature range from 40 to 550 °C.
